# Functional Brain Network Disruptions in Parkinson’s Disease: Insights from Information Theory and Machine Learning

**DOI:** 10.3390/diagnostics14232728

**Published:** 2024-12-04

**Authors:** Ömer Akgüller, Mehmet Ali Balcı, Gabriela Cioca

**Affiliations:** 1 Faculty of Science, Department of Mathematics, Mugla Sitki Kocman University, Muğla 48000, Turkey; oakguller@mu.edu.tr; 2 Engineering Sciences Department, Engineering and Architecture Faculty, Izmir Katip Celebi University, Izmir 35620, Turkey; 3 Preclinical Department, Faculty of Medicine, Lucian Blaga University of Sibiu, 550024 Sibiu, Romania; gabriela.cioca@ulbsibiu.ro

**Keywords:** Parkinson’s disease, brain network, information theory, explainable deep learning

## Abstract

**Objectives:** This study investigates disruptions in functional brain networks in Parkinson’s Disease (PD), using advanced modeling and machine learning. Functional networks were constructed using the Nonlinear Autoregressive Distributed Lag (NARDL) model, which captures nonlinear and asymmetric dependencies between regions of interest (ROIs). Key network metrics and information-theoretic measures were extracted to classify PD patients and healthy controls (HC), using deep learning models, with explainability methods employed to identify influential features. **Methods:** Resting-state fMRI data from the Parkinson’s Progression Markers Initiative (PPMI) dataset were used to construct NARDL-based networks. Metrics, such as Degree, Closeness, Betweenness, and Eigenvector Centrality, along with Network Entropy and Complexity, were analyzed. Convolutional Neural Networks (CNNs), Recurrent Neural Networks (RNNs), and Long Short-Term Memory (LSTM) models, classified PD and HC groups. Explainability techniques, including SHAP and LIME, identified significant features driving the classifications. **Results:** PD patients showed reduced Closeness (22%) and Betweenness Centrality (18%). CNN achieved 91% accuracy, with Network Entropy and Eigenvector Centrality identified as key features. Increased Network Entropy indicated heightened randomness in PD brain networks. **Conclusions:** NARDL-based analysis with interpretable deep learning effectively distinguishes PD from HC, offering insights into neural disruptions and potential personalized treatments for PD.

## 1. Introduction

Parkinson’s Disease (PD) is a chronic, progressive neurodegenerative disorder characterized predominantly by motor symptoms, such as bradykinesia, rigidity, resting tremor, and postural instability, which result from the loss of dopaminergic neurons in the substantia nigra pars compacta of the midbrain [1,2]. While these motor manifestations are the most visible and have historically been the primary focus for diagnosis and treatment, PD is increasingly recognized as a multisystem disorder that affects various non-motor domains, including cognitive, psychiatric, autonomic, and sensory functions [3,4].

Cognitive impairments in PD range from mild cognitive impairment to Parkinson’s Disease Dementia and significantly impact patients’ quality of life and daily functioning [5,6]. Understanding the neural mechanisms underlying cognitive decline in PD is crucial for the early detection, prognosis, and the development of targeted therapeutic interventions [7].

Functional connectivity refers to the temporal correlations and statistical dependencies between spatially remote neurophysiological events, which provide insights into the coordinated activity of different brain regions [8]. Functional Magnetic Resonance Imaging (fMRI) has emerged as a pivotal tool for investigating functional connectivity, leveraging blood-oxygen-level-dependent (BOLD) signals to infer neural activity [9,10]. Resting-state fMRI allows for the examination of intrinsic brain networks without task-induced confounds, offering a window into the brain’s functional organization in both health and disease [11,12].

Graph-theoretical approaches have been widely applied to fMRI data to construct functional brain networks, where nodes represent regions of interest (ROIs) and edges represent functional connections based on statistical associations [13,14]. These methods enable the quantification of network properties such as efficiency, modularity, and centrality, facilitating the exploration of how brain networks reorganize in PD [15,16]. Studies have reported alterations in network topology in PD patients, including decreased global efficiency, disrupted small-world properties, and altered connectivity within specific networks like the default mode network and the frontoparietal network [17,18,19]. These network changes are associated with both motor and cognitive symptoms, suggesting that functional connectivity alterations underpin the clinical manifestations of PD [20,21].

However, traditional graph-theoretical analyses often rely on linear correlation measures, such as Pearson’s correlation, to estimate functional connectivity [8]. While these methods are valuable, they may not fully capture the complex, nonlinear, and dynamic interactions within the brain, particularly in pathological conditions like PD [22]. Neural systems exhibit both linear and nonlinear dependencies, and their interactions can be asymmetric and time-varying, especially in PD [23]. Therefore, advanced analytical techniques that can model these complexities are essential for a more accurate and comprehensive understanding of functional connectivity alterations in PD.

This study aims to bridge this gap by employing the Nonlinear Autoregressive Distributed Lag (NARDL) model to construct functional brain networks from resting-state fMRI data of PD patients and healthy controls (HC). By capturing both nonlinear and asymmetric dependencies between ROIs, the NARDL model provides a nuanced representation of neural interactions that traditional linear models may overlook. Additionally, the study integrates information-theoretic measures to quantify the informational properties of brain networks, offering deeper insights into the structural and functional disruptions caused by PD. Coupled with explainable deep learning models, this approach not only enhances the detection and classification of PD but also ensures that the findings are interpretable and clinically relevant. These advancements contribute to the development of more accurate diagnostic tools and personalized treatment strategies, ultimately improving patient outcomes and advancing our understanding of PD’s impact on brain connectivity.

This study is structured as follows: Section 2 reviews the existing literature on functional connectivity in PD, highlighting gaps such as the underutilization of nonlinear modeling and explainable machine learning. Section 3 details the participant cohort, data acquisition, application of the NARDL model for constructing functional brain networks, and the extraction of connectivity and information-theoretic measures. Section 4 presents the findings from statistical analyses and deep learning models; detailed discussions interpreting these results are provided immediately afterward within the same section to contextualize and understand their implications. Finally, Section 5 summarizes the key contributions of the study, emphasizing how integrating NARDL-based modeling, information-theoretic measures, and explainable deep learning advances our understanding of functional brain network alterations in PD and can inform clinical practices.

## 2. Related Works

In Parkinson’s disease, functional connectivity studies reveal significant insights into both motor and non-motor symptoms, including cognitive decline. Resting-state fMRI studies, for instance, have consistently shown altered connectivity within the default mode network, sensorimotor networks, and other brain regions, correlating these changes with cognitive and motor impairments in PD patients [24,25,26]. These findings suggest that disruptions in functional networks may serve as early indicators of disease progression, making functional connectivity a valuable biomarker for tracking neurodegeneration.

Studies using whole-brain functional connectivity analyses highlight reduced connectivity in regions associated with cognition and motor control, such as the fronto-striatal and visual networks, which often precede notable cognitive and functional deficits [27,28]. Additionally, dynamic functional connectivity, which measures the temporal fluctuations of functional connectivity, has shown that patients with advanced cognitive impairment in PD tend to experience a higher prevalence of segregated network states, limiting inter-network communication necessary for cognitive flexibility [29,30].

Research by Ref. [27] illustrated abnormal connectivity patterns in PD patients, identifying compensatory increases in connectivity in the posterior cingulate cortex and precuneus, which are linked to the brain’s efforts to maintain functional stability despite neurodegeneration. Studies by Refs. [29,30] have demonstrated a correlation between reduced functional connectivity in networks like the default mode network and cognitive impairments, suggesting functional connectivity as a potential marker for PD-related cognitive decline.

The application of functional connectivity in diagnostic classifications has achieved high accuracy, as shown by studies using machine learning on whole-brain connectivity, which differentiate PD patients from controls and correlate network changes with disease severity [31,32,33].

Studying functional connectivity in PD not only illuminates the underlying network disruptions associated with cognitive and motor impairments but also supports the potential for FC as an early diagnostic and prognostic marker in tracking disease progression. Foundational studies have established FC alterations as integral to understanding PD pathology, especially regarding non-motor symptoms that significantly affect patient quality of life.

Graph theory provides a framework to quantitatively analyze brain network topology, revealing the organization of functional networks and aiding in the understanding of complex neurological disorders like Parkinson’s disease (PD). This approach enables the use of specific metrics, such as degree centrality, clustering coefficient, path length, and small-world properties, to characterize the brain’s network organization and the alterations induced by PD.

For instance, degree centrality reflects the number of connections a node has, indicating its importance in the network. In PD, reduced centrality has been observed in regions related to motor and cognitive processing, highlighting disrupted communication in critical areas [34]. The clustering coefficient measures the degree to which nodes in a network tend to cluster together, often reflecting local efficiency. In PD, clustering often decreases, indicating reduced local connectivity and integration [35,36]. Regarding the average distance between nodes, where shorter path lengths suggest efficient information transfer, PD studies reveal increased path length, indicating disrupted global efficiency in brain networks [37,38]. While both PD patients and healthy controls typically exhibit small-world properties, PD often demonstrates a shift toward more random, less efficient networks [39,40,41,42].

Brain connectivity is inherently dynamic and influenced by numerous nonlinear dependencies between regions. Traditional linear models, such as static correlation, are limited in capturing this complexity and may overlook crucial aspects of brain interactions. Nonlinear methods, like the nonlinear autoregressive distributed lag model and mutual information, are better suited for uncovering these complex dependencies. These methods allow for a more nuanced analysis, capturing subtle variations in connectivity patterns that are temporally and spatially heterogeneous.

Ref. [43] utilized dynamic graph measures, including the Fiedler value, to assess time-dependent network changes in PD. Their findings showed lower variability in modularity and global efficiency in PD patients, indicating a more rigid network organization. This rigidity was linked to disease severity, underscoring how nonlinear, dynamic metrics offer a deeper understanding of PD pathology. Ref. [44] observed that patients with higher depression severity exhibited weakened interhemispheric connections and decreased clustering in brain networks based on mutual information, especially between parietal–occipital and frontal regions. This reduction in connectivity was associated with greater depressive symptoms, suggesting that mutual information-based brain network metrics could serve as biomarkers for assessing depression in post-stroke patients. Using cross-mutual information in a time-frequency domain, Ref. [45] decodes how brain regions communicate during grasp tasks. This approach is crucial for understanding motor control and coordination, which could aid in developing interventions for motor impairments. Ref. [46] combines symbolic mutual information and Kolmogorov-Arnold Complexity features from EEG data to improve early Parkinson’s diagnosis. These methods enhance EEG signal analysis, allowing for more accurate differentiation between healthy and early-stage Parkinson’s patients.

Information-theoretic measures such as Shannon entropy, network complexity, and Integrated Information Theory (IIT) provide powerful tools to assess functional brain networks by quantifying aspects like randomness, complexity, and integration within neural systems. These metrics are crucial in understanding the network disruptions that characterize neurodegenerative diseases like PD, where cognitive impairment and motor dysfunctions often correlate with altered brain connectivity.

Ref. [47] introduces an efficient method leveraging entropy measures on resting-state EEG (rs-EEG) data for diagnosing and tracking Parkinson’s disease (PD). By comparing various entropy methods, the study found fuzzy entropy to be the most effective, achieving 99.9% classification accuracy in distinguishing PD patients from healthy controls. Ref. [48] comprehensively reviewed and suggested that Alzheimer’s patients generally exhibit increased regularity and reduced entropy in EEG signals, suggesting a decline in brain signal complexity. Studies using methods like Approximate Entropy and Sample Entropy reveal significant differences in parietal and occipital regions when compared to healthy controls. Ref. [49] suggested that applying entropy and fractional analysis methods to electrophysiological recordings can lead to a deeper understanding of the pathophysiology of neurodegenerative diseases, which are linked to changes in brain activity complexity. Specifically, it suggests that Alzheimer’s disease is associated with a decrease in global brain activity complexity, while Parkinson’s disease shows a localized increase in brain signal complexity. Ref. [50] emphasized that understanding how the brain processes information for cognition requires recognizing the different types of information involved—namely unique, redundant, and synergistic elements. It highlights that information decomposition techniques are instrumental in distinguishing these components, reshaping our understanding of integrative brain function and its neural organization. Ref. [50] also reviewed evidence integrating structural, molecular, and functional underpinnings of redundancy and synergy, exploring their roles in cognition, computation, and their evolutionary development, suggesting that this framework is key to comprehending the brain’s informational architecture.

Deep learning has gained prominence in analyzing functional connectivity data to enhance the diagnosis and prognosis of Parkinson’s disease. By leveraging sophisticated neural networks, researchers can identify disease-specific connectivity patterns and predict clinical outcomes, facilitating early diagnosis and targeted interventions. Ref. [51] used brain graph convolutional networks to classify PD patients by modeling EEG-derived functional connectivity data as a graph. This model achieved high precision (95.59%), emphasizing its efficacy in distinguishing PD from control subjects by retaining spatial interdependence among EEG channels. CNNs, often combined with transfer learning, have been applied to MRI and fMRI datasets to classify PD patients. Ref. [52] employed CNNs with data augmentation and achieved an accuracy of 89.23%, indicating that these models can effectively distinguish structural and functional alterations in PD. Ref. [53] presented a study on the early detection of Parkinson’s disease using MRI and deep learning, comparing 2D and 3D CNN models trained on pre-processed MRI scans. The results show that the 3D CNN significantly outperforms the 2D model, achieving 88.9% accuracy with 0.86 AUC, making it more reliable for identifying key features of the disease. Ref. [54] provides a bibliometric analysis and literature review of research on Parkinson’s Disease (PD) diagnosis using deep learning (DL), highlighting the advancements in the field. The study, based on papers from the Scopus database, shows strong development in DL-based PD diagnosis worldwide while identifying a research gap in incremental learning approaches for big data analysis.

While previous research has significantly advanced our understanding of functional brain network alterations in Parkinson’s Disease, several limitations persist. The predominant reliance on linear models fails to capture the nonlinear and asymmetric dynamics of neural connectivity inherent in PD. The underutilization of information-theoretic measures limits insights into the brain’s informational and integrative capacities affected by the disease. Moreover, the “black box” nature of deep learning models poses challenges for clinical adoption due to a lack of interpretability. Methodological shortcomings, such as small sample sizes and cross-sectional designs, further constrain the generalizability and applicability of findings.

Our study aims to address these gaps by integrating several advanced methodologies into a cohesive framework. By employing the NARDL model, we capture both nonlinear and asymmetric dependencies between brain regions, providing a more accurate and nuanced representation of functional connectivity alterations in PD. This approach allows us to model the complex temporal dynamics and directionality of interactions that traditional linear models may overlook.

## 3. Materials and Methods

In the current study, we aimed to bridge the gap in PD research by integrating NARDL-based functional connectivity modeling with information-theoretic measures and explainable deep learning models. We apply the NARDL model to resting-state fMRI data to construct functional brain networks that capture both linear and nonlinear, as well as asymmetric dependencies between ROIs. This approach allows us to generate Combined Dependency (CD) values, reflecting the intricate temporal relationships and interactions within the brain. From these networks, a comprehensive set of connectivity measures is extracted, as well as information-theoretic metrics, including Degree Centrality, Closeness Centrality, Betweenness Centrality, Eigenvector Centrality, Network Entropy, Network Complexity, and IIT.

Our participant cohort consists of HC individuals and PD patients, enabling us to investigate the functional connectivity alterations associated with PD. By employing deep learning models—specifically CNNs, RNNs, and LSTMs—it is aimed to classify HC and PD subjects based on the extracted features. These models are selected for their ability to handle high-dimensional data and capture complex patterns within the connectivity measures. The CNN model leverages spatial hierarchies in the data, the RNN captures sequential dependencies, and the LSTM is adept at modeling long-term dependencies, reflecting different aspects of brain functional connectivity.

To interpret the models’ predictions and understand the underlying mechanisms, SHAP and LIME are utilized. SHAP provides a global interpretation by quantifying the average contribution of each feature across all predictions, highlighting which features consistently influence the model’s decisions [55]. LIME offers a local, instance-specific interpretation, revealing how feature contributions vary for individual predictions and capturing the heterogeneity within the PD group. This combination of techniques allows us to identify key network features that differentiate PD from HC, providing insights into the neural underpinnings of PD.

**Figure 1:**
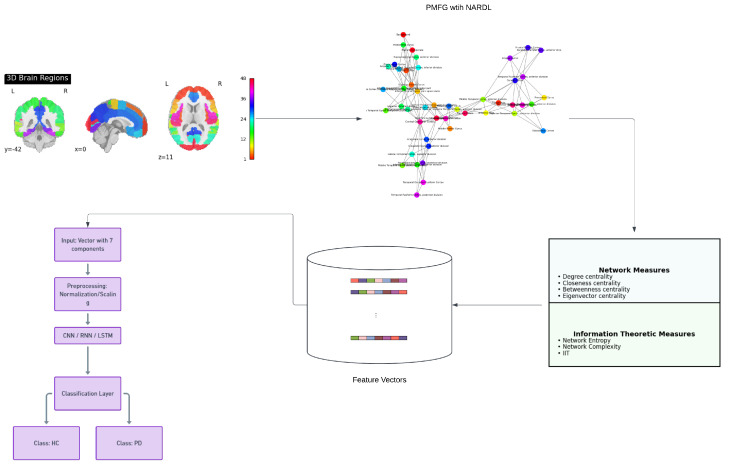
Outline of the methodology.

The outline of the methodology of this study is presented in Figure 1.

### 3.1. Dataset

The Parkinson’s Progression Markers Initiative (PPMI) dataset represents a pivotal resource in the study of Parkinson’s Disease, offering a comprehensive and meticulously curated collection of clinical and neuroimaging data aimed at identifying biomarkers for disease progression [56]. In this study, the PPMI dataset is specifically utilized to examine functional connectivity patterns through fMRI. The dataset comprises a substantial cohort of participants, categorized into two primary groups: Healthy Controls (HC) and individuals diagnosed with Parkinson’s Disease. Among the collected samples, there are 142 HC data points and a significantly larger subset of 1537 PD data points, reflecting the extensive efforts to capture the heterogeneity and progression of PD within the study population.

The selection of fMRI as the modality for data analysis is strategic, given its unparalleled ability to non-invasively measure and map brain activity by detecting changes associated with blood flow. This technique provides high-resolution spatial and temporal insights into the neural dynamics underlying cognitive and motor functions, which are often disrupted in Parkinson’s Disease. By focusing on fMRI data, the study leverages advanced imaging techniques to construct detailed brain networks, facilitating the exploration of functional connectivity and its alterations in PD compared to healthy individuals. The rich fMRI data allow for the identification of specific ROIs and the examination of their interconnections, thereby uncovering the intricate patterns of neural interactions that may serve as indicators of disease progression or therapeutic response.

The substantial imbalance in the dataset, with 1537 PD samples compared to 142 HC samples, underscores the prevalence and research emphasis on Parkinson’s Disease within the PPMI initiative. This disparity necessitates the application of robust statistical and machine learning techniques to address potential biases and ensure that the classification models developed are both accurate and generalizable. Techniques such as Synthetic Minority Over-sampling Technique (SMOTE) are employed to balance the class distribution, thereby enhancing the model’s ability to distinguish between HC and PD groups effectively. The extensive number of PD samples provides a rich foundation for training deep learning models, enabling the capture of subtle and complex patterns in brain connectivity that may differentiate diseased states from healthy ones.

### 3.2. Network Measures

Brain networks serve as the intricate scaffolding through which the brain orchestrates its diverse functions, encompassing cognition, emotion, and motor control. Mathematically, these networks are adeptly modeled using graph theory, wherein each brain region is represented as a node $V=\{v_{1},v_{2},\ldots ,v_{n}\}$ and the connections between them as edges $E=\{e_{ij}\}$, with $e_{ij}$ denoting the interaction between nodes $v_{i}$ and $v_{j}$. Unlike unweighted networks, where edges merely indicate the presence or absence of a connection, weighted networks incorporate an additional layer of information by assigning a weight $w_{ij}$ to each edge, quantifying the strength or intensity of the connection between the corresponding nodes. This weighted adjacency matrix $W\in R^{n\times n}$ thus captures both the topology and the nuanced interaction strengths within the network, providing a more granular and informative representation of brain connectivity.

The analysis of weighted brain networks involves a suite of network metrics that delve into various structural and functional aspects of the brain’s connectivity architecture. In this study, four key metrics are studied: Weighted Degree Centrality, Weighted Clustering Coefficient, Weighted Eigenvector Centrality, and Weighted Closeness Centrality, each providing unique insights into the network’s organization and dynamics.

Weighted Degree Centrality extends beyond the mere count of connections to encapsulate the cumulative strength of interactions emanating from a node. For a given node $v_{i}$, the Weighted Degree Centrality $C_{D}^{w}\left(v_{i}\right)$ is mathematically defined as

(1) $$C_{D}^{w}\left(v_{i}\right)=\sum_{j=1}^{n}w_{ij},$$

where $w_{ij}$ represents the weight of the edge between nodes $v_{i}$ and $v_{j}$, and *n* is the total number of nodes in the network. This metric quantifies the total connectivity strength of $v_{i}$, highlighting regions that act as hubs with extensive and robust connections facilitating widespread communication across the brain.

Closeness Centrality quantifies how close a node is to all other nodes in the network, reflecting its ability to quickly interact with the entire network. In weighted networks, the Weighted Closeness Centrality $C_{Cl}^{w}\left(v_{i}\right)$ for node $v_{i}$ is defined as

(2) $$C_{Cl}^{w}\left(v_{i}\right)=\frac{1}{\sum_{j\neq i}d^{w}\left(v_{i},v_{j}\right)},$$

where $d^{w}\left(v_{i},v_{j}\right)$ represents the Weighted Shortest Path Length between nodes $v_{i}$ and $v_{j}$. The weighted shortest path length is often computed by treating the weights as inverse measures of connection strength or as direct measures of traversal cost, commonly defined as

(3) $$d^{w}\left(v_{i},v_{j}\right)=\min_{\mathrm{paths}}\sum_{(u,v)\in \mathrm{path}}\frac{1}{w_{uv}}.$$

Here, higher weights $w_{uv}$ imply stronger and more efficient connections, thereby reducing the effective path length. The Weighted Closeness Centrality thus reflects the overall efficiency of information transfer from $v_{i}$ to all other nodes in the network, with higher values indicating a more central and integrative position within the brain network.

Betweenness centrality is a fundamental measure in network analysis that quantifies the importance of a node in terms of its role as an intermediary in the communication paths between other nodes. In weighted networks, where edges have associated weights representing strength, cost, or capacity, the calculation of betweenness centrality takes these weights into account, altering the paths considered for the centrality measure. For a given node *v* in a weighted network G=(V,E,w), where *V* is the set of nodes, *E* is the set of edges, and w(e) is the weight of edge *e*, the betweenness centrality BC(v) is defined by

(4) $$C_{B}^{w}\left(v\right)=\sum_{s\neq v\neq t}\frac{\sigma_{st}\left(v\right)}{\sigma_{st}}.$$

In this equation, $\sigma_{st}$ denotes the total number of shortest paths from node *s* to node *t*, while $\sigma_{st}\left(v\right)$ is the number of those paths that pass through node *v*. The centrality of node *v* thus measures how frequently it appears on these shortest paths relative to all node pairs (s,t) in the network.

Eigenvector Centrality assesses the influence of a node within the network by considering not only its direct connections but also the importance of the nodes it is connected to. The Weighted Eigenvector Centrality $C_{E}^{w}\left(v_{i}\right)$ for node $v_{i}$ is defined implicitly by the eigenvalue equation

(5) $$C_{E}^{w}\left(v_{i}\right)=\frac{1}{\lambda }\sum_{j=1}^{n}w_{ij}C_{E}^{w}\left(v_{j}\right).$$

In this equation, λ represents the largest eigenvalue of the weighted adjacency matrix and $C_{E}^{w}\left(v_{j}\right)$ is the eigenvector centrality of node $v_{j}$. This metric measures the influence of a node in the network, where a high eigenvector centrality indicates that the node is connected to other highly influential nodes, thereby playing a significant role in information dissemination and network stability.

Entropy in the context of weighted networks is a measure that quantifies the level of disorder or uncertainty associated with the distribution of weights and connectivity within the network. It serves as an indicator of the network’s complexity and the diversity of interactions. The concept of entropy can be used to understand the variability in edge weights, the distribution of node connections, and the overall structure of the network.

For a weighted network G=(V,E,w), where *V* is the set of nodes, *E* is the set of edges, and w(e) represents the weight of an edge *e*, the entropy H(G) can be defined based on the distribution of edge weights. One common approach is to treat the weights as a probability distribution over the edges and compute the Shannon entropy. Let P(e) be the normalized weight of edge *e*, defined by

(6) $$P\left(e\right)=\frac{w(e)}{\sum_{e^{\prime }\in E}w\left(e^{\prime }\right)},$$

where $\sum_{e^{\prime }\in E}w\left(e^{\prime }\right)$ is the total sum of all edge weights in the network, ensuring that P(e) represents a valid probability distribution.

The entropy H(G) of the network can then be expressed as

(7) $$H\left(G\right)=-\sum_{e\in E}P\left(e\right)\log P\left(e\right)$$

This equation reflects the amount of uncertainty or diversity in the distribution of edge weights. A higher value of H(G) indicates that the edge weights are more evenly distributed, suggesting a higher level of disorder and more diverse interactions between nodes. Conversely, a lower entropy implies that the weights are concentrated on a few edges, indicating that the network has more structured or homogeneous connections.

These network metrics collectively provide a comprehensive understanding of the structural properties and functional organization of the network. Weighted Degree Centrality highlights key nodes that maintain significant overall connectivity within the network. Closeness Centrality measures the efficiency with which a node can communicate with all other nodes, offering insights into information flow and network integration. Betweenness Centrality pinpoints critical nodes that act as bridges, facilitating communication between different network regions and thus influencing network resilience and connectivity. Eigenvector Centrality identifies influential nodes that are connected to other well-connected nodes, revealing the hierarchical importance of nodes within the network. Network Entropy captures the distributional complexity of weights and connections, providing an assessment of the network’s overall structural diversity and robustness.

### 3.3. Brain Network Formation

In the initial phase of this study, comprehensive brain networks are meticulously constructed by leveraging fMRI data obtained from the PPMI datasets. The study focuses on participants categorized into distinct cognitive states, specifically Healthy Controls (HC) and Parkinson’s Disease (PD). Recognizing the critical importance of standardized anatomical references, the Harvard-Oxford Atlas is employed to parcellate the brain into predefined Regions of Interest (ROIs), ensuring anatomical consistency across all subjects.

The construction of the brain networks involved several computational steps, leveraging the Harvard-Oxford Atlas to provide a standardized anatomical framework. Specifically, the cort-maxprob-thr25-2mm version of the atlas is utilized, which includes cortical regions with a probability threshold of 25%, resampled to 2 mm isotropic resolution. This atlas allowed us to parcellate the brain into 48 ROIs for each participant, ensuring consistency across all subjects. The use of the Harvard-Oxford Atlas facilitated the extraction of time series data from anatomically defined cortical areas, which is crucial for accurate and reproducible network analyses. Data acquisition begins with the conversion of DICOM files to NIfTI format using the dcm2niix tool, facilitating seamless integration with subsequent analysis pipelines. Each participant’s fMRI data undergoes rigorous preprocessing, including motion correction to eliminate artifacts from head movements, spatial normalization to align individual brain images to the Montreal Neurological Institute space, and temporal filtering to isolate relevant frequency bands while attenuating noise and physiological fluctuations. Following these preprocessing steps, the NiftiLabelsMasker from the Nilearn library is utilized to extract mean fMRI signal time series for each ROI based on the Harvard-Oxford Atlas, resulting in discrete sequences of measurements that reflect neural activity over the scanning period.

Subsequent to time series extraction, the study employs the NARDL model to quantify the dependencies between each pair of ROIs. For two discrete time series representing different ROIs, the NARDL approach captures both short-run and long-run dependencies by modeling the relationships through positive and negative partial sums of the time series’ differences. Specifically, for each pair of ROIs, the NARDL model is fitted with a specified number of lags (e.g., two lags) to estimate the influence of past values of one ROI on the current values of another. Mathematically, let $X_{t}$ and $Y_{t}$ represent the time series of two ROIs at time *t*. The NARDL model can be expressed as

(8) $$Y_{t}=\alpha +\sum_{i=1}^{p}\phi_{i}Y_{t-i}+\sum_{i=0}^{p}\beta_{i}^{+}X_{t-i}^{+}+\sum_{i=0}^{p}\beta_{i}^{-}X_{t-i}^{-}+\epsilon_{t},$$

where α is the intercept term, *p* is the number of lags, $X_{t}^{+}=\max\left(\Delta X_{t},0\right)$ captures the positive changes in *X*, $X_{t}^{-}=\min\left(\Delta X_{t},0\right)$ captures the negative changes in *X*, $\phi_{i}$ are the coefficients for the lagged dependent variable, $\beta_{i}^{+}$ and $\beta_{i}^{-}$ are the coefficients for the positive and negative partial sums of the independent variable, respectively, $\epsilon_{t}$ is the error term.

The short-run dependencies are represented by the coefficients $\beta_{i}^{+}$ and $\beta_{i}^{-}$, while the long-run dependencies are derived from these coefficients in conjunction with the autoregressive terms $\phi_{i}$. Specifically, the long-run positive and negative dependencies can be calculated as

(9) $$\begin{array}{ccc} \mathrm{Long}-\mathrm{run}\;\mathrm{positive}\;\mathrm{dependency}(LP) & = & \frac{\sum_{i=0}^{p}\beta_{i}^{+}}{1-\sum_{i=1}^{p}\phi_{i}} \end{array}$$

(10) $$\begin{array}{ccc} \mathrm{Long}-\mathrm{run}\;\mathrm{negative}\;\mathrm{dependency}(LN) & = & \frac{\sum_{i=0}^{p}\beta_{i}^{-}}{1-\sum_{i=1}^{p}\phi_{i}}. \end{array}$$

The combined dependency between ROIs *X* and *Y* is then computed by aggregating the short-run and long-run positive dependencies and subtracting the aggregated short-run and long-run negative dependencies

(11) $$\mathrm{Combined}\;\mathrm{Dependency}\left(CD\right)=\left|\left(\mathrm{SP}+\mathrm{LP}\right)-\left(\mathrm{SN}+\mathrm{LN}\right)\right|,$$

where SP and SN denote short-run positive and short-run negative, respectively. This formulation ensures that the dependency measure accounts for both the positive and negative influences over different temporal scales, providing a robust metric of functional connectivity that captures the nuanced interactions inherent in neural dynamics.

The utilization of NARDL models in this study offers several significant advantages that enhance the robustness and depth of the functional connectivity analysis. By simultaneously capturing short-term fluctuations and long-term equilibrium relationships between ROIs, NARDL provides a comprehensive understanding of the dynamic interactions within the brain. This dual capability is particularly valuable in neurological studies where both immediate responses and sustained patterns of connectivity are of interest. Additionally, the ability of NARDL to differentiate between positive and negative changes allows for a more nuanced interpretation of how increases or decreases in neural activity in one region influence another, thereby uncovering asymmetric dependencies that traditional linear models might overlook. Moreover, NARDL’s flexibility in accommodating nonlinear relationships ensures that complex, non-proportional interactions inherent in brain networks are accurately modeled, leading to more precise and meaningful connectivity measures. This methodological strength is crucial for identifying subtle yet significant alterations in brain connectivity associated with Parkinson’s Disease, facilitating the detection of biomarkers that could inform diagnosis and therapeutic strategies.

Upon computing the dependencies for all possible ROI pairs, a symmetric weighted adjacency matrix $W\in R^{n\times n}$ is constructed, where *n* denotes the number of ROIs and each element $W_{ij}$ corresponds to the dependency value between ROIs $v_{i}$ and $v_{j}$

(12) $$W_{ij}=\mathrm{Combined}\;\mathrm{Dependency}\left(X_{i},Y_{j}\right).$$

To ensure comparability across subjects and mitigate inter-subject variability in overall connectivity strength, the dependency values within each adjacency matrix are normalized using min-max scaling

(13) $$W_{ij}^{\prime }=\frac{W_{ij}-\min\left(W\right)}{\max(W)-\min(W)},$$

where $W_{ij}^{\prime }$ represents the normalized dependency value. This scaling facilitates the subsequent application of network filtration techniques by ensuring that connectivity measures are on a consistent scale across all subjects.

The resultant normalized weighted adjacency matrix $W^{\prime }$ embodies the complete brain network for each individual, capturing the strength and complexity of functional interactions between ROIs. To distill the most significant connections while preserving the network’s structural integrity, Planar Maximally Filtered Graph (PMFG) filtration is applied. This process retains the most influential edges in the network, ensuring planarity and thereby simplifying the network’s topology for further analysis. The final PMFG-filtered network serves as the foundation for subsequent analyses, including comparisons between cognitive groups and the exploration of network topology alterations associated with Parkinson’s Disease.

### 3.4. Brain Network Filtration

PMFG serves as a sophisticated network filtering technique designed to distill the most significant connections within the brain network while preserving its fundamental topological properties [57,58,59,60]. Mathematically, the PMFG process begins by considering the weighted adjacency matrix $W^{\prime }\in R^{n\times n}$, where each element $W_{ij}^{\prime }$ represents the normalized CD value between ROIs $v_{i}$ and $v_{j}$. The objective is to construct a planar graph $G_{PMFG}=\left(V,E_{PMFG}\right)$ that retains a maximal subset of edges from $W^{\prime }$ without violating the planarity constraint, which dictates that the graph can be embedded in a two-dimensional plane without any edges crossing.

The filtration process commences by sorting all potential edges *E* in descending order based on their CD weights $W_{ij}^{\prime }$. Formally, the edge set *E* is ordered so that $W_{i_{1}j_{1}}^{\prime }\geq W_{i_{2}j_{2}}^{\prime }\geq \ldots \geq W_{i_{m}j_{m}}^{\prime }$, where $m=\frac{n(n-1)}{2}$. Starting with an empty graph, edges are iteratively added from the sorted list to $G_{PMFG}$ provided their inclusion does not violate the planarity condition. This is mathematically enforced by ensuring that for any new edge $e_{ij}$ being considered, the augmented graph $G^{\prime }=G_{PMFG}\cup \left\{e_{ij}\right\}$ remains planar. The planarity of $G^{\prime }$ can be verified using Kuratowski’s theorem, which states that a graph is planar if and only if it does not contain a subgraph that is a subdivision of $K_{5}$ (the complete graph on five vertices) or $K_{3,3}$ (the complete bipartite graph on two sets of three vertices) [61].

The culmination of the PMFG filtration process results in a planar graph $G_{PMFG}$ that encapsulates the most robust and significant CD-based connections within the brain network while adhering to the topological constraints of planarity. This graph retains a greater number of edges compared to simpler filtering methods like the Minimum Spanning Tree, thereby preserving more of the network’s intrinsic modular and hierarchical structures.

### 3.5. Information Theoretic Measures

Upon establishing the foundational brain networks through Combined Dependency estimation and PMFG filtration, the subsequent phase of this study delves into the quantitative evaluation of these networks using a suite of advanced information-theoretic and network complexity measures. This multifaceted analysis encompasses the calculation of network entropies, the application of Integrated Information Theory (IIT) metrics, and the positioning of networks within the Complexity-Entropy (H-C) plane, each of which provides a unique lens through which to assess the structural and functional intricacies of brain connectivity in Parkinson’s disease.

The computation of network entropies begins with the determination of Shannon entropy, a fundamental measure that quantifies the uncertainty or randomness inherent in the distribution of connection strengths across the brain network. Given a weighted adjacency matrix $W^{\prime }\in R^{n\times n}$, where each element $W_{ij}^{\prime }$ represents the normalized CD between regions $v_{i}$ and $v_{j}$, the Shannon entropy H(G) of the network *G* is defined as

(14) $$H\left(G\right)=-\sum_{i=1}^{n}\sum_{j=1}^{n}p_{ij}\log p_{ij}$$

here, $p_{ij}$ is the probability associated with the edge $e_{ij}$, calculated by normalized CD values

(15) $$p_{ij}=\frac{W_{ij}^{\prime }}{\sum_{k=1}^{n}\sum_{l=1}^{n}W_{kl}^{\prime }}$$

This normalization ensures that the probabilities sum to one, allowing the entropy to effectively capture the distribution of connection strengths. Higher values of H(G) indicate a more heterogeneous distribution, suggesting greater complexity and diversity in network connectivity, whereas lower values imply a more uniform and potentially less adaptable network structure.

In tandem with entropy measures, the study incorporates IIT metrics to assess the degree of information integration within the brain network. IIT posits that the level of consciousness or information integration Φ within a system is a function of how interconnected and interdependent its components are. Mathematically, Φ is conceptualized as the difference between the total information generated by the whole system and the sum of information generated by its disjoint parts

(16) $$\Phi =I\left(G\right)-\sum_{m}I\left(G_{m}\right),$$

where I(G) represents the integrated information of the entire network *G*, and $I(G_{m})$ denotes the information of each subsystem $G_{m}$ when the network is partitioned. The precise computation of Φ involves intricate algorithms that partition the network into its constituent modules, calculate the information content of each partition, and evaluate the loss of integrated information upon such partitioning. This measure provides deep insights into the network’s capacity for unified information processing, which is critical in understanding the disruptions caused by Parkinson’s disease.

To simplify the networks and focus on the most significant connections, each normalized adjacency matrix is converted into a binary network. A thresholding method is applied, where any connection weight equal to or exceeding a specified fraction (θ) of the maximum weight in the matrix is set to one, and all other connections are set to zero

(17) $$b_{ij}=\left\{\begin{array}{cc} 1, & \mathrm{if}\;p_{ij}\geq \theta \cdot p_{\max},\\ 0, & \mathrm{otherwise}, \end{array}\right.$$

where $p_{\max}=\max_{i,j}p_{ij}$ and θ is set to 0.001 in this study. This process reduces computational complexity by focusing on the strongest connections and eliminates self-connections by setting the diagonal elements to zero.

The integrated information Φ is computed following the principles of IIT, as outlined in Equation (16). The total information I(G) of the whole network is calculated using the Shannon entropy formula given in Equation (14) applied to the probability distribution derived from the binary adjacency matrix.

For each bipartition, the information content $I(G_{m})$ of each subsystem is calculated

(18) $$I\left(G_{m}\right)=-\sum_{i,j\in G_{m}}p_{ij}^{\left(G_{m}\right)}\log p_{ij}^{\left(G_{m}\right)},$$

where $p_{ij}^{\left(G_{m}\right)}$ are the normalized probabilities within subsystem $G_{m}$. The integrated information for a given bipartition is then

(19) $$\Phi_{\mathrm{partition}}=I\left(G\right)-\sum_{m}I\left(G_{m}\right).$$

Due to the exponential growth of possible bipartitions with network size—specifically, $2^{n-1}-1$ for a network with *n* nodes—exhaustive computation of Φ across all bipartitions is computationally infeasible for large networks. To address this challenge, an approximate method using random sampling of bipartitions is employed.

For each network, an approximate value of Φ is computed by randomly sampling a fixed number (N = 10,000) of bipartitions. In each sample, a subset size *k* is randomly selected from the range 1 to n−1, where *n* is the number of nodes. A subset *S* of *k* nodes is randomly selected, and the complement subset $S^{\prime }$ is defined as $S^{\prime }=V\setminus S$, where *V* is the set of all nodes.

For each bipartition, the information content $I(G_{S})$ and $I(G_{S^{\prime }})$ of the subsystems corresponding to subsets *S* and $S^{\prime }$ are calculated using the submatrices derived from the binary adjacency matrix. The probabilities $p_{ij}^{\left(S\right)}$ and $p_{ij}^{\left(S^{\prime }\right)}$ within each subsystem are normalized by dividing the connection weights by the total weight within that subsystem

(20) $$p_{ij}^{\left(S\right)}=\frac{b_{ij}^{\left(S\right)}}{\sum_{i,j\in S}b_{ij}^{\left(S\right)}},\;p_{ij}^{\left(S^{\prime }\right)}=\frac{b_{ij}^{\left(S^{\prime }\right)}}{\sum_{i,j\in S^{\prime }}b_{ij}^{\left(S^{\prime }\right)}}.$$

The integrated information for each bipartition is then computed as

(21) $$\Phi_{\mathrm{partition}}=I\left(G\right)-\left(I\left(G_{S}\right)+I\left(G_{S^{\prime }}\right)\right).$$

The overall integrated information of the network is estimated by averaging the $\Phi_{\mathrm{partition}}$ values over all sampled bipartitions

(22) $$\Phi \approx \frac{1}{N}\sum_{i=1}^{N}\Phi_{\mathrm{partition},i}.$$

This method provides a practical approximation of Φ without the need for exhaustive enumeration, enabling the analysis of large networks with a high number of nodes.

Further enriching the analysis, the H-C plane offers a graphical representation that juxtaposes the entropy H(G) against a measure of statistical complexity C(G). Statistical complexity is defined as the product of entropy and disequilibrium D(G), where disequilibrium quantifies the deviation of the network’s probability distribution from uniformity

(23) C(G)=H(G)×D(G).

Here, disequilibrium D(G) is often calculated as the Euclidean distance between the network’s probability distribution $p_{ij}$ and a uniform distribution $p_{ij}^{\mathrm{uniform}}=\frac{1}{n^{2}}$

(24) $$D\left(G\right)=\sqrt{\sum_{i=1}^{n}\sum_{j=1}^{n}{\left(p_{ij}-p_{ij}^{\mathrm{uniform}}\right)}^{2}}.$$

By plotting C(G) against H(G), the H-C plane elucidates the balance between randomness and structured complexity within the brain network. Networks that lie toward higher entropy and complexity regions are indicative of intricate and adaptable connectivity patterns, while those positioned toward lower entropy and complexity suggest more rigid and less diverse interactions. In the context of Parkinson’s disease, shifts in the placement of brain networks on the H-C plane can reveal alterations in the balance between integration and segregation of neural processes, thereby shedding light on the disease’s impact on cognitive and motor functions.

### 3.6. Explainable Deep Learning

This study employs a comprehensive computational framework to analyze and classify brain network alterations associated with Parkinson’s Disease using deep learning models and explainable artificial intelligence (XAI) techniques.

The study begins by loading feature vectors composed of means of $C_{D}^{w}$, $C_{Cl}^{w}$, $C_{B}^{w}$, and $C_{E}^{w}$; and information theoretic measures H(G), C(G), and Φ, all extracted from combined dependency network data of HC $X_{\mathrm{HC}}\in R^{n_{\mathrm{HC}}\times d}$ and PD $X_{\mathrm{PD}}\in R^{n_{\mathrm{PD}}\times d}$ subjects. Here, $n_{\mathrm{HC}}$ and $n_{\mathrm{PD}}$ denote the number of HC and PD subjects, respectively, and *d* represents the number of features extracted from each subject.

The feature matrices are concatenated to form the combined dataset

(25) $$X=\left[\begin{array}{c} X_{\mathrm{HC}}\\ X_{\mathrm{PD}} \end{array}\right],\;y=\left[\begin{array}{c} 0_{n_{\mathrm{HC}}}\\ 1_{n_{\mathrm{PD}}} \end{array}\right],$$

where $y\in {\left\{0,1\right\}}^{n}$ is the label vector indicating class membership, with $n=n_{\mathrm{HC}}+n_{\mathrm{PD}}$.

To ensure that each feature contributes equally to the model, the study standardizes the features using z-score normalization

(26) $${\tilde{x}}_{ij}=\frac{x_{ij}-\mu_{j}}{\sigma_{j}},\;\forall i\in \left\{1,\ldots ,n\right\},\;\forall j\in \left\{1,\ldots ,d\right\},$$

where $\mu_{j}$ and $\sigma_{j}$ are the mean and standard deviation of feature *j* across all subjects.

Due to potential class imbalance (i.e., $n_{\mathrm{HC}}\neq n_{\mathrm{PD}}$), the Synthetic Minority Over-sampling Technique (SMOTE) is applied [62] to the training data to create a balanced dataset. SMOTE generates synthetic samples for the minority class by interpolating between existing minority class samples. For each minority class sample $x_{i}$, the study identifies its *k* nearest neighbors within the minority class. Synthetic samples are generated using

(27) $$x_{\mathrm{synthetic}}=x_{i}+\delta \left(x_{\mathrm{NN}}-x_{i}\right),$$

where $x_{\mathrm{NN}}$ is a randomly selected neighbor, and δ∼U(0,1) is a random scalar drawn from a uniform distribution.

In this study, three types of deep learning models CNN, RNN, and LSTM are utilized. Each model processes the input features to perform binary classification.

The CNN model applies convolutional filters to capture local patterns in the data. For input reshaped to $X\in R^{d\times 1}$, the convolution operation is defined as

(28) $$h^{\left(1\right)}=\sigma^{\left(1\right)}\left(W^{\left(1\right)}*X+b^{\left(1\right)}\right),$$

where ∗ denotes convolution, and the remaining layers are similar to the MLP.

The RNN is designed to capture sequential dependencies. Treating the input features as a sequence ${\left\{x_{t}\right\}}_{t=1}^{d}$, the hidden state at time step *t* is updated as

(29) $$h_{t}=\sigma_{h}\left(W_{xh}x_{t}+W_{hh}h_{t-1}+b_{h}\right),$$

with $h_{0}$ initialized appropriately. The output is derived from the final hidden state $h_{d}$.

The LSTM network extends the capabilities of traditional RNNs by incorporating specialized gating mechanisms that effectively manage long-term dependencies and mitigate issues such as the vanishing gradient problem. Specifically, the LSTM architecture introduces three gates: the forget gate, the input gate, and the output gate, each playing a crucial role in regulating the flow of information through the network. For input features treated as a sequence ${\left\{x_{t}\right\}}_{t=1}^{d}$, the LSTM updates its cell state $c_{t}$ and hidden state $h_{t}$ at each time step *t* using the following equations

(30) $$\begin{array}{cc} f_{t} & =\sigma_{f}\left(W_{f}x_{t}+U_{f}h_{t-1}+b_{f}\right), \end{array}$$

(31) $$\begin{array}{cc} i_{t} & =\sigma_{i}\left(W_{i}x_{t}+U_{i}h_{t-1}+b_{i}\right), \end{array}$$

(32) $$\begin{array}{cc} o_{t} & =\sigma_{o}\left(W_{o}x_{t}+U_{o}h_{t-1}+b_{o}\right), \end{array}$$

(33) $$\begin{array}{cc} g_{t} & =\tanh\left(W_{g}x_{t}+U_{g}h_{t-1}+b_{g}\right), \end{array}$$

(34) $$\begin{array}{cc} c_{t} & =f_{t}⊙c_{t-1}+i_{t}⊙g_{t}, \end{array}$$

(35) $$\begin{array}{cc} h_{t} & =o_{t}⊙\tanh\left(c_{t}\right), \end{array}$$

where $\sigma_{f}$, $\sigma_{i}$, and $\sigma_{o}$ denote the sigmoid activation functions for the forget gate, input gate, and output gate, respectively, and ⊙ represents element-wise multiplication. The forget gate $f_{t}$ determines the extent to which information from the previous cell state $c_{t-1}$ should be retained, effectively allowing the network to forget irrelevant or outdated information. The input gate $i_{t}$ controls the incorporation of new information into the cell state, modulated by $g_{t}$, which generates candidate values for updating the cell state. Finally, the output gate $o_{t}$ regulates the information flow from the cell state to the hidden state $h_{t}$, determining which aspects of the cell state are exposed to the next layer or the output.

Stratified *k*-Fold Cross-Validation with k=10 is employed to ensure robust model evaluation. The training objective is to minimize the binary cross-entropy loss

(36) $$L\left(\theta \right)=-\frac{1}{N}\sum_{i=1}^{N}\left[y_{i}\log{\hat{y}}_{i}+\left(1-y_{i}\right)\log\left(1-{\hat{y}}_{i}\right)\right],$$

where θ denotes the model parameters, and *N* is the number of training samples. Optimization is performed using stochastic gradient descent with appropriate learning rates and regularization techniques (e.g., dropout, $L_{2}$ regularization).

Model performance is evaluated using the following metrics

(37) $$\begin{array}{cc} \mathrm{Accuracy} & =\frac{TP+TN}{TP+TN+FP+FN}, \end{array}$$

(38) $$\begin{array}{cc} \mathrm{Precision} & =\frac{TP}{TP+FP}, \end{array}$$

(39) $$\begin{array}{cc} \mathrm{Recall}\;\left(\mathrm{Sensitivity}\right) & =\frac{TP}{TP+FN}, \end{array}$$

(40) $$\begin{array}{cc} F1-\mathrm{Score} & =2\cdot \frac{\mathrm{Precision}\times \mathrm{Recall}}{\mathrm{Precision}+\mathrm{Recall}}, \end{array}$$

where TP, TN, FP, and FN represent true positives, true negatives, false positives, and false negatives, respectively.

To interpret the predictions of the deep learning models, SHAP and LIME explanations are employed. SHAP values assign an importance value $\phi_{j}$ to each feature *j* for a particular prediction, based on the concept of Shapley values from cooperative game theory. The SHAP value for feature *j* is calculated as

(41) $$\phi_{j}=\sum_{S\subseteq F\setminus \{j\}}\frac{|S|!(|F|-|S|-1)!}{|F|!}\left(f_{S\cup \{j\}}\left(x_{S\cup \{j\}}\right)-f_{S}\left(x_{S}\right)\right),$$

where F is the set of all features, *S* is a subset of features not containing *j*, $f_{S}$ is the model trained on features in *S*, and $x_{S}$ is the input restricted to features in *S*.

LIME approximates the complex model locally around a prediction using an interpretable model, typically a linear model

(42) $$\hat{f}\left(x\right)=\beta_{0}+\sum_{j=1}^{d}\beta_{j}x_{j},$$

where $\beta_{j}$ are coefficients estimated by minimizing a loss function weighted by a proximity measure $\pi_{x}$

(43) $$\beta =\arg\min_{\beta }\sum_{i=1}^{N}\pi_{x}\left(x_{i}\right){\left(f\left(x_{i}\right)-\hat{f}\left(x_{i}\right)\right)}^{2}+\lambda {∥\beta ∥}_{1},$$

with λ being a regularization parameter.

To obtain global feature importance, we aggregate LIME explanations across multiple instances. For each feature *j*, we compute the mean and standard deviation of its coefficients

(44) $$\begin{array}{cc} {\bar{\beta }}_{j} & =\frac{1}{M}\sum_{i=1}^{M}\beta_{ij}, \end{array}$$

(45) $$\begin{array}{cc} \sigma_{j} & =\sqrt{\frac{1}{M}\sum_{i=1}^{M}{\left(\beta_{ij}-{\bar{\beta }}_{j}\right)}^{2}}, \end{array}$$

where $\beta_{ij}$ is the LIME coefficient for feature *j* in instance *i*, and *M* is the total number of instances.

#### Hyperparameters

In this study, the deep learning models are meticulously implemented using TensorFlow and Keras [63], providing robust frameworks for constructing and training complex neural networks essential for analyzing high-dimensional brain imaging data. Data manipulation and analysis are performed using NumPy and Pandas, which offer efficient handling of large datasets and facilitate preprocessing steps such as normalization and feature selection. To address the issue of class imbalance inherent in medical datasets, the SMOTE is applied using the imbalanced-learn library [64]. SMOTE generates synthetic examples of the minority class by interpolating between existing minority class samples, thereby enhancing the model’s ability to learn from underrepresented patterns. For model interpretability, SHAP and LIME are employed, leveraging their respective Python packages to generate insights into feature importance and model decision processes, which is crucial in a clinical context.

In the CNN model, the optimal hyperparameters were determined to be an optimizer of adam, a kernel size of 5, 32 filters, 50 epochs, a dropout rate of 0.3, and a batch size of 16. The selection of the Adam optimizer facilitated efficient and adaptive learning by adjusting the learning rates during training, which is particularly beneficial when dealing with complex and high-dimensional data like the network features extracted from the adjacency matrices. The kernel size of 5 allowed the model to capture broader patterns across the input features, effectively recognizing spatial hierarchies within the connectivity data. Utilizing 32 filters struck a balance between model complexity and computational efficiency, providing sufficient capacity to learn diverse feature representations without overfitting. Training the model for 50 epochs ensured adequate exposure to the data for learning while preventing excessive training that could lead to overfitting. The dropout rate of 0.3 introduced regularization by randomly omitting 30% of the neurons during training, which helped in mitigating overfitting by preventing the co-adaptation of neurons. Lastly, a batch size of 16 was chosen to ensure that each update step had enough data to make meaningful progress in learning while keeping memory usage manageable, which is essential given the resource constraints.

For the RNN model, the best hyperparameters identified included 64 units in the recurrent layer, the adam optimizer, 50 epochs, a dropout rate of 0.3, and a batch size of 16. The use of 64 units allowed the RNN to maintain a sufficient memory capacity to capture sequential dependencies within the data, which is critical when modeling time series or sequential features derived from network connectivity. Employing the Adam optimizer once again facilitated adaptive learning rates, enhancing the convergence speed and stability during training. Training over 50 epochs provided the model with enough iterations to learn the underlying patterns without overfitting. A dropout rate of 0.3 served as a regularization technique to reduce overfitting by randomly deactivating neurons during training, thus encouraging the model to develop redundant representations that generalize better to new data. The batch size of 16 offered a practical compromise between stochastic and batch gradient descent, ensuring that each training step was computationally efficient and that the gradient estimates were stable enough for effective learning.

In the LSTM network, the optimal hyperparameters were found to be 128 units in the LSTM layer, the adam optimizer, 50 epochs, a dropout rate of 0.4, and a batch size of 16. The increased number of units, compared to the RNN model, provided the LSTM with enhanced capacity to learn long-term dependencies and complex temporal dynamics inherent in the network features. This is particularly important given the LSTM’s ability to capture patterns over extended sequences, which is valuable for interpreting intricate connectivity structures in brain networks. The use of the Adam optimizer continued to offer adaptive learning rates and efficient optimization, contributing to the model’s effective training process. The model was trained over 50 epochs to ensure sufficient learning while avoiding overfitting. A higher dropout rate of 0.4 was employed to address the increased risk of overfitting due to the larger model size, enhancing the model’s generalization capabilities by preventing reliance on any single neuron or path through the network. The batch size of 16 remained consistent with the other models, maintaining an effective balance between computational efficiency and the statistical reliability of gradient estimates during training.

By parametrically designing these models with considerations for architecture depth, activation functions, regularization techniques, and optimization algorithms, the study aims to effectively capture the underlying patterns associated with Parkinson’s Disease from complex brain imaging data. The combination of deep learning models and interpretability methods like SHAP and LIME facilitates not only accurate classification but also provides insights into the most significant features contributing to the predictions, thereby enhancing the potential for clinical applicability and understanding of the disease mechanisms.

## 4. Results and Discussions

In the ensuing subsections, the results and discussions pertaining to emerging mean network measures and information-theoretic measures are presented. These analyses are conducted initially from a statistical perspective and subsequently from a deep learning perspective.

### 4.1. Statistical Perspective

**Figure 2:**
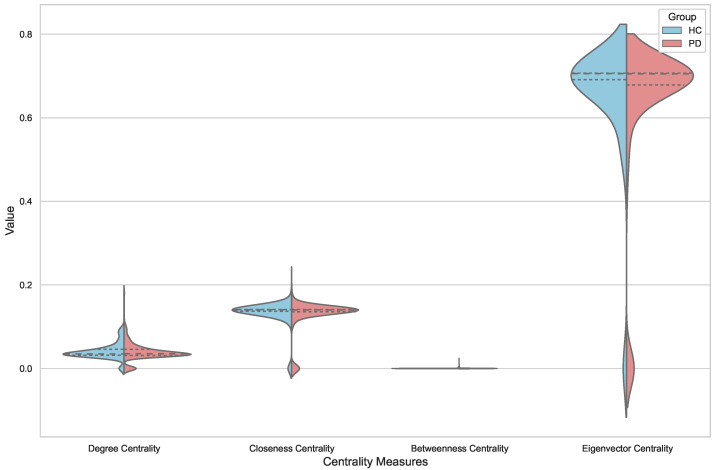
Violin plots showing the distribution of average values for brain network metrics across Healthy Control (HC) and Parkinson’s Disease (PD) groups.

Violin plots in Figure 2 illustrate the distributions of mean values for four key network metrics—Degree Centrality, Closeness Centrality, Betweenness Centrality, and Eigenvector Centrality—across two brain network conditions: Healthy Control (HC) and Parkinson’s Disease (PD) groups. These metrics provide insights into the topological characteristics of brain networks, highlighting how the brain’s network structure may evolve from a cognitively healthy state to one associated with neurodegenerative changes like those observed in Parkinson’s Disease.

Starting with Degree Centrality, the distributions for both HC and PD groups appear relatively similar, with slight variations in the median values and the spread of the data. This similarity suggests that, on average, the strength of connections—quantified as the sum of the weights of connections at each node—in the networks of HC and PD subjects may not differ substantially. However, the violin plot reveals subtle differences in the density distribution, hinting at potential outliers or slight variations in node connectivity strength within each group. These nuances could be important, as they might reflect individual differences in how brain regions are interconnected in healthy versus diseased states.

In contrast, the Closeness Centrality plots for HC and PD reveal more pronounced differences. The distributions indicate that, although both groups have a central peak in similar regions, the spread is slightly wider for the PD group. This wider spread could imply that individuals with Parkinson’s Disease exhibit more variability in how “close” a node is to all other nodes in terms of the average shortest path lengths. This variability might reflect disruptions in efficient communication pathways within brain networks due to the disease, which lead to alterations in functional integration. The increased spread in the PD group could be indicative of heterogeneous disease progression or compensatory mechanisms attempting to maintain network efficiency despite pathological changes.

The Betweenness Centrality distributions for both groups show an interesting pattern: very narrow plots with minimal density, indicating that this measure is consistently low across both HC and PD subjects. This consistency suggests that the role of nodes as intermediaries in the shortest paths between other nodes is relatively limited in these brain networks. Moreover, the minimal differentiation between the two groups concerning this specific centrality measure suggests that Betweenness Centrality may not be significantly affected by the neurodegenerative processes in Parkinson’s Disease or that it is not a sensitive marker for distinguishing between healthy and diseased brain networks in this context.

The most striking difference is observed in the Eigenvector Centrality plots. Here, the distributions for the HC and PD groups show a marked contrast, with the HC distribution being more concentrated around a central value, while the PD distribution spreads much wider across a range of values. This indicates a significant difference in the influence of nodes within the networks. In the PD group, nodes might exhibit greater variability in how connected they are to other highly connected nodes. The broad distribution for PD suggests that certain regions of the brain may have enhanced or reduced influence in network communication compared to the HC group. This variability could be due to the disease’s effect on neural pathways and functional connectivity, possibly leading to some nodes becoming more central in compensatory networks, while others lose their centrality due to degeneration.

**Figure 3:**
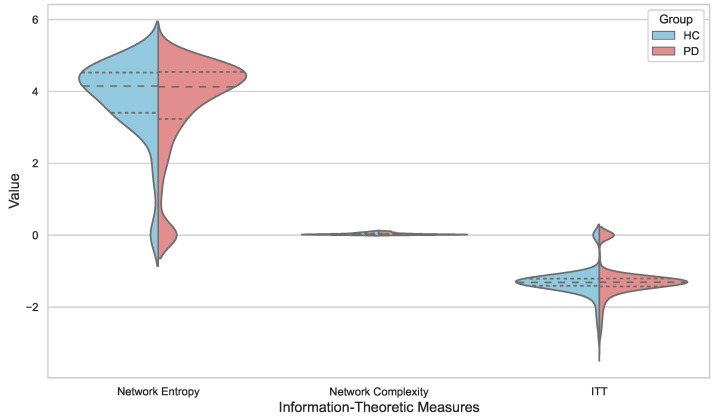
Violin plots showing the distribution of information-theoretic measures across Healthy Control (HC) and Parkinson’s Disease (PD) groups.

Figure 3 provides violin plots illustrating the distribution of information-theoretic measures—specifically Network Entropy, Network Complexity, and IIT—for both HC and PD groups. These plots allow for a comparative analysis of how these metrics vary between the two groups, offering insights into potential differences in the complexity and informational properties of brain networks in healthy and diseased states.

The plot for Network Entropy demonstrates notable differences between the HC and PD groups. Both distributions have a similar overall shape, but the HC group appears to have a slightly more dispersed distribution compared to the PD group, which exhibits a more concentrated range around the median. This suggests that while the average entropy—reflecting the randomness or unpredictability of the brain network connections—is somewhat consistent across both groups, the HC group shows greater variability. This variability in entropy might indicate that healthy brains have a wider range of complexity levels, potentially related to adaptive or dynamic responses in neural connectivity. The PD group’s relatively more uniform distribution could imply a certain level of homogeneity in brain network entropy, potentially due to reduced flexibility or altered neural connectivity patterns characteristic of the disease.

In terms of Network Complexity, both groups display minimal differences, with only slight variations. The violin plots show that both the HC and PD groups have similarly low values, with minor deviations in their spread. This uniformity could suggest that this specific measure may not strongly differentiate between HC and PD brain networks or that it is less sensitive to the connectivity changes induced by Parkinson’s Disease. The minimal spread also hints at a consistent structural property across both healthy and diseased brain networks, possibly indicating that network complexity remains relatively stable despite neurodegenerative changes.

The distribution for IIT reveals some distinctions between the two groups. The violin plot indicates that while the distributions of IIT values are centered similarly for both HC and PD groups, the HC group exhibits a more condensed shape, suggesting a tighter clustering of IIT values. The PD distribution, on the other hand, shows a slightly broader range, indicating more variability in how information integration and differentiation occur within the PD brain networks. This variability could reflect disruptions in how efficiently information is processed or integrated—a hallmark of Parkinson’s Disease as it affects cognitive and motor functions. The broader distribution for the PD group suggests that IIT could potentially be an informative measure for distinguishing subtle changes in brain network processing and structural integration in PD patients.

**Figure 4:**
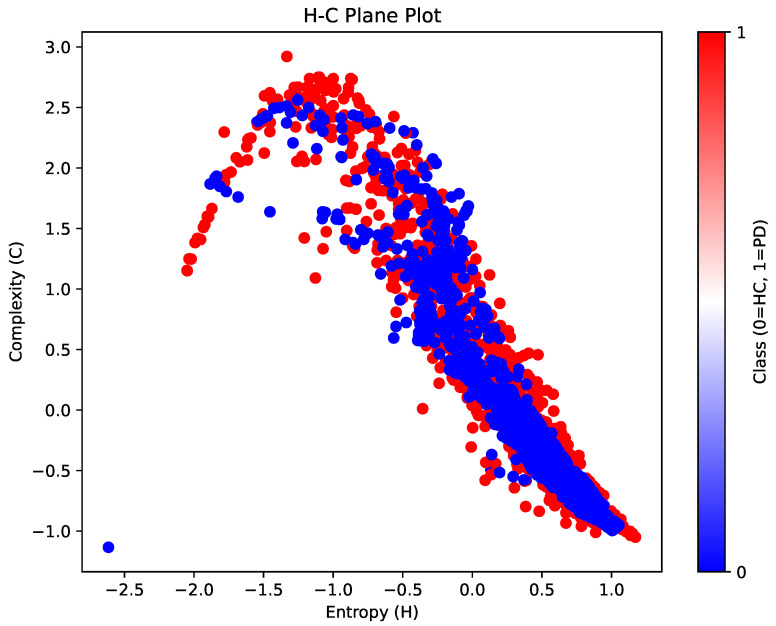
H-C Plane across Healthy Control (HC) and Parkinson’s Disease (PD) groups.

Figure 4 presents the H-C Plane plot, mapping the relationship between entropy (H) and complexity (C) for brain networks across HC and PD groups. The HC group is represented in blue, while the PD group is shown in red, allowing for a visual comparison of how these two measures vary between the groups. The H-C Plane is a valuable visualization as it provides insights into how randomness (entropy) and structural complexity coexist in brain networks, informing us about the network’s stability, efficiency, and adaptability.

Figure 4 reveals an intriguing distribution pattern between the HC and PD groups. For both groups, there is a generally negative correlation between entropy and complexity, consistent with the theoretical notion that systems with higher entropy typically exhibit lower structural complexity and vice versa. However, the distributions show that the PD group tends to have higher complexity values than the HC group at equivalent entropy levels. This is reflected by the broader spread and denser clustering of red points (PD group) in the upper complexity region of the plot.

This suggests that, in PD patients, brain networks may exhibit more pronounced structural organization despite having varying levels of randomness. Such an increase in structural complexity in PD could be indicative of compensatory mechanisms or pathological network changes associated with the disease. The PD group’s wider spread in complexity might reflect network reorganization efforts involving changes in both short-term and long-term dependencies.

The distribution of the blue points (HC group) is more concentrated along a lower complexity range with a relatively wider spread in entropy. This implies that healthy brain networks might maintain a balance between randomness and complexity, indicative of an adaptable and efficient system capable of dynamically responding to external stimuli. The higher entropy levels in the HC group suggest greater variability and flexibility in neural connectivity, essential for normal cognitive functioning.

The NARDL model captures both short-run and long-run dependencies between pairs of Regions of Interest (ROIs), aggregated into a robust metric reflecting combined influences over time. The CD measures provide a nuanced view of functional connectivity, capturing directional and asymmetric interactions within the brain. Integrating this dependency information into adjacency matrices and processing it into network features like entropy and complexity enriches the analysis by incorporating temporal dynamics that might otherwise be overlooked.

In the context of the H-C Plane, the higher complexity observed in the PD group likely reflects network reorganization efforts involving changes in both short-term and long-term dependencies. The CD values derived from NARDL analyses contribute to these elevated complexity levels, indicating that PD networks might rely more on long-term compensatory connections or exhibit altered interactions that increase structural organization but at the cost of adaptability. The PD distributions covering a wider range of complexity suggest that disease-related changes in neural dynamics are more variable, possibly due to differences in disease severity, progression rates, or the brain’s heterogeneous response to degeneration.

In what follows in this subsection, statistical tests are presented after applying SMOTE to address the significant class imbalance present in the dataset. The original dataset comprises 1537 Parkinson’s Disease samples compared to only 142 Healthy Control samples, which poses challenges for reliable statistical analysis. Class imbalance can lead to biased results, reduce the statistical power of tests, and increase the likelihood of Type I and Type II errors, thereby obscuring true differences between groups. By implementing SMOTE, synthetic samples are generated for the minority class (HC), effectively balancing the class distribution. This balancing enhances the fairness and sensitivity of statistical tests, ensuring that comparisons between HC and PD groups are not disproportionately influenced by the overwhelming number of PD samples. Consequently, SMOTE facilitates a more accurate and equitable assessment of the underlying differences in network measures between the two groups. Additionally, balancing the classes allows for more robust effect size estimations and clearer insights into the relationships between variables. However, it is important to acknowledge that while SMOTE mitigates class imbalance, it introduces synthetic data, which may not fully capture the natural variability of the minority class, potentially affecting the authenticity of the results. Therefore, the application of SMOTE in this context is carefully considered to enhance the validity of statistical analyses while being mindful of its limitations.

The Shapiro–Wilk test is a statistical method used to assess the normality of a dataset by comparing the data’s distribution to a theoretically normal distribution. The null hypothesis for the Shapiro–Wilk test states that the data are normally distributed. If the *p*-value obtained from the test is less than the chosen significance level (commonly 0.05), the null hypothesis is rejected, indicating that the data do not follow a normal distribution. This test is particularly useful for verifying the appropriateness of parametric statistical methods, which often assume normality in the data.

The Shapiro–Wilk test for normality conducted on the network metrics for both the HC and PD groups after applying SMOTE demonstrates in Table 1 that the data for all tested metrics remains significantly non-normal. The test statistics for each metric are consistently below 1, and the *p*-values are either extremely small or effectively zero, indicating a strong rejection of the null hypothesis of normality. For example, in the HC group, Degree Centrality has a test statistic of 0.8371 with a *p*-value of $5.24\times 10^{-37}$, confirming a significant deviation from normality. Similar results are observed for metrics like Closeness Centrality, Betweenness Centrality, and Eigenvector Centrality, where the *p*-values are effectively zero, emphasizing highly non-normal distributions.

The results are consistent across both groups, suggesting that the application of SMOTE, which is used to balance the data between HC and PD groups by synthetically generating examples in the minority class, does not alter the underlying distributional characteristics of the features. This finding highlights that while SMOTE is useful for balancing class representation in a dataset, it does not modify the shape or normality of the feature distributions. The persistence of non-normality in all tested metrics after SMOTE has implications for the choice of statistical methods in subsequent analysis. Parametric tests that assume normal distributions, such as t-tests or ANOVA, are not suitable for these data. Instead, non-parametric methods, such as the Mann–Whitney U test, should be employed to ensure valid results when comparing metrics between the HC and PD groups.

Levene’s test for homogeneity of variance is a statistical method used to determine whether the variances of different groups are equal. This test is particularly useful when comparing the assumption of equal variances between groups in preparation for statistical tests that assume homogeneity of variance. The null hypothesis for Levene’s test states that the variances across groups are equal. If the *p*-value is less than the significance level (typically 0.05), the null hypothesis is rejected, indicating unequal variances between groups.

The results of Levene’s test in Table 2 for homogeneity of variance after applying SMOTE reveal that for most network metrics, the null hypothesis of equal variances between the HC and PD groups is rejected. Metrics such as Degree Centrality, Closeness Centrality, Betweenness Centrality, Eigenvector Centrality, Network Entropy, and IIT have *p*-values that are significantly below 0.05. For example, Degree Centrality shows a test statistic of 15.6362 with a *p*-value of $7.85\times 10^{-5}$, and IIT exhibits an even more pronounced result with a statistic of 27.7121 and a *p*-value of $1.50\times 10^{-7}$. These results suggest that the assumption of homogeneity of variance does not hold for these metrics, indicating significant differences in variances between the HC and PD groups.

On the other hand, Network Complexity presents a *p*-value of 0.1294, which is above the 0.05 threshold, suggesting that the null hypothesis of equal variances cannot be rejected for this metric. This indicates that the variances for Network Complexity are similar between the HC and PD groups, maintaining the homogeneity of variance assumption for this specific feature.

The Mann–Whitney U test is a non-parametric statistical test used to determine whether there are significant differences between the distributions of two independent groups. This test is particularly suitable when the data do not meet the assumptions of normality or homogeneity of variance, as identified in the previous analyses. The null hypothesis of the Mann–Whitney U test states that the distributions of the two groups are the same. A *p*-value less than the chosen significance level (e.g., 0.05) indicates that the null hypothesis should be rejected, implying a significant difference between the groups.

The Mann–Whitney U test results in Table 3 for network metrics after applying SMOTE indicate significant differences between the HC and PD groups for specific metrics, while others do not show significant differences. Closeness Centrality and Betweenness Centrality both have *p*-values that fall below the standard significance threshold, even after adjustment for multiple comparisons. Closeness Centrality has an adjusted *p*-value of 0.0098, while Betweenness Centrality shows a highly significant result with an adjusted *p*-value of $6.60\times 10^{-6}$. This indicates that there are meaningful distributional differences in these metrics between the HC and PD groups. The effect sizes for these two features, −0.0665 for Closeness Centrality and −0.1021 for Betweenness Centrality, suggest that the differences, while statistically significant, are moderate in magnitude.

For other metrics, such as Degree Centrality, Network Complexity, and IIT, the results are not significant after adjusting for multiple comparisons, as indicated by their adjusted *p*-values being greater than 0.05. For instance, Degree Centrality has an adjusted *p*-value of 0.6450, and IIT shows no significant difference with an adjusted *p*-value of 1.0. The effect sizes for these metrics are also minimal, indicating negligible practical differences between the groups. Eigenvector Centrality, Network Entropy, and IIT, in particular, exhibit very small effect sizes (e.g., −0.0096 for Eigenvector Centrality), reinforcing the conclusion that the distributions of these metrics between HC and PD are similar.

The presence of statistically significant results for some features but not others suggests that certain network metrics are more sensitive to the differences between the HC and PD groups. Betweenness Centrality and Closeness Centrality, for example, may reflect more distinct structural or functional network properties related to brain connectivity in Parkinson’s Disease. In contrast, metrics such as IIT and Eigenvector Centrality do not appear to provide strong differentiation between the two groups in this analysis.

The Bonferroni correction is a statistical method used to adjust *p*-values for multiple comparisons to control for Type I error (false positives). This correction involves multiplying the original *p*-value by the number of comparisons made, thus making it more stringent to achieve significance. The aim is to ensure that the probability of making at least one Type I error remains below a chosen significance level, typically 0.05.

After applying the Bonferroni correction, only Closeness Centrality and Betweenness Centrality remain statistically significant in Table 4. The adjusted *p*-values for these metrics are 0.0098 and $6.60\times 10^{-6}$, respectively, indicating that the differences between the HC and PD groups for these features are robust and unlikely to be due to random chance. This suggests that Closeness Centrality and Betweenness Centrality may be particularly relevant metrics for differentiating between these two groups, potentially highlighting underlying differences in network properties, such as how central or mediating certain nodes are within the brain network.

For the other metrics, the Bonferroni correction has led to adjusted *p*-values that exceed the significance threshold of 0.05. For instance, Degree Centrality has an adjusted *p*-value of 0.6450, and Network Complexity is at 0.2398, both of which indicate non-significant differences between the HC and PD groups after accounting for multiple comparisons. Metrics such as Eigenvector Centrality, Network Entropy, and IIT show even higher adjusted *p*-values of 1.0, underscoring that these features do not provide significant differentiation between the groups under the stricter criteria of the Bonferroni correction. The correction highlights that while initial *p*-values might indicate significance, adjusting for multiple tests is crucial for maintaining the reliability of results. The fact that only two features remain significant after correction suggests that Closeness Centrality and Betweenness Centrality could be more sensitive to the changes in brain network structure associated with Parkinson’s Disease. This underscores the importance of these metrics in network analysis and their potential role in characterizing differences in brain connectivity.

The comparison between the tables reveals key insights into the statistical behavior of network metrics derived from the NARDL-based formation of networks for the HC and PD groups. The Shapiro–Wilk test results consistently show that all network metrics for both groups are non-normally distributed, even after SMOTE was applied to balance the class sizes. This outcome suggests that the complex and nuanced network formations obtained using NARDL, which capture both short-term and long-term dependencies between ROIs, inherently produce metrics that do not conform to normal distributions. The non-normality observed across metrics such as Degree Centrality, Closeness Centrality, and Betweenness Centrality reflects the intricate dependencies modeled by NARDL that characterize brain connectivity in both healthy and diseased states. Levene’s test for homogeneity of variance indicated that most network metrics do not meet the assumption of equal variances between the HC and PD groups, with the exception of Network Complexity. The significant *p*-values for metrics such as Closeness Centrality and Betweenness Centrality highlight the variance differences captured in the NARDL-based network structure. This suggests that these specific metrics are sensitive to the heterogeneity present in brain connectivity, potentially reflecting the effects of Parkinson’s Disease on network stability and interaction strength across different brain regions. The Mann–Whitney U test provided a detailed look at which metrics showed significant distributional differences between HC and PD groups. Initially, Closeness Centrality and Betweenness Centrality were found to be significant, reinforcing their role as key metrics that can differentiate between the two groups based on the network structure derived from NARDL. However, after applying the Bonferroni correction, which adjusts for multiple comparisons to reduce the risk of Type I errors, only these two metrics remained significant. This outcome highlights their robustness and reliability in detecting meaningful differences when stringent significance criteria are applied. The Bonferroni correction results provide a final layer of comparison by showing how the significance of the metrics changes when correcting for multiple comparisons. While the Mann–Whitney U test suggested initial significance for some metrics, only Closeness Centrality and Betweenness Centrality retained their significance post-correction. This adjustment emphasizes that the detected differences in these metrics are not due to chance, reinforcing the value of these specific measures derived from the NARDL-based network formations in understanding brain network alterations in Parkinson’s Disease.

**Figure 5:**
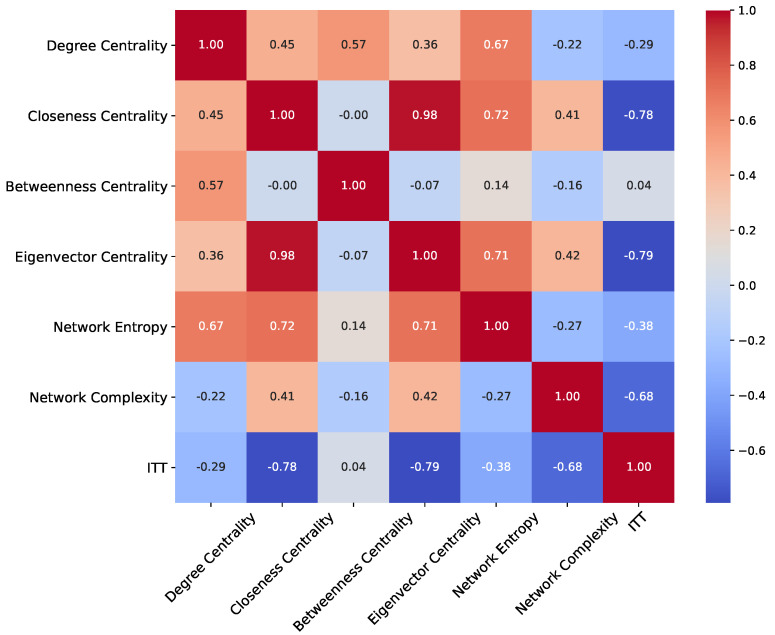
Correlation matrix of network metrics for Healthy Control (HC) and Parkinson’s Disease (PD) groups.

The correlation matrix of network measures after applying SMOTE in Figure 5 reflects the intricate relationships between the metrics derived from the NARDL-based network formation and sheds light on how these relationships might differ between the HC and PD classes. NARDL, which models both short-term and long-term dependencies between ROIs, contributes to a deeper understanding of functional connectivity and the derived metrics that capture different aspects of this connectivity. The results observed in the correlation matrix suggest that the way these metrics interrelate may highlight structural and functional differences in the brain connectivity of HC and PD groups.

The strong positive correlation between Closeness Centrality and Eigenvector Centrality (0.98) implies that these centrality metrics share a similar role in characterizing the central and influential nodes within the brain’s network. In the context of the NARDL-based formation, this correlation suggests that brain regions that are centrally positioned (with higher Closeness Centrality) are also likely connected to other highly influential regions (indicated by high Eigenvector Centrality). This relationship could be more pronounced or vary in complexity between HC and PD groups, where PD networks may exhibit changes in how these central regions interact due to disruptions in connectivity. The dependency structures captured by NARDL might reveal how centrality shifts from healthy to diseased states, with certain regions losing or gaining prominence in PD, leading to potential changes in this strong correlation.

Network Entropy’s positive correlation with both Degree Centrality (0.67) and Closeness Centrality (0.72) reflects that networks with more extensive node connections and central nodes are associated with higher levels of randomness or connectivity complexity. From a NARDL-based perspective, this relationship highlights how dependencies between brain regions contribute to overall network entropy, potentially indicating how randomness in connectivity patterns might differ in HC and PD groups. In PD, changes in the short-term and long-term relationships between ROIs could alter how entropy correlates with centrality measures. For example, disrupted or reorganized connectivity in PD might lead to a shift in how these metrics align, suggesting that PD networks may have altered the balance between connectivity strength and entropy compared to HC networks.

The negative correlation involving IIT (e.g., −0.78 with Closeness Centrality and −0.79 with Eigenvector Centrality) is particularly intriguing when considering the NARDL-based network formation. IIT captures the network’s integrated and differentiated information processing capabilities, and its inverse relationship with centrality measures suggests that as networks become more integrated in their information processing, they may rely less on central nodes or specific pathways. In PD, this relationship could indicate that as the disease progresses, the brain’s ability to process information in an integrated manner is compromised, potentially leading to a reduction in the importance of central nodes and an increase in distributed network processing. The NARDL-based model, which incorporates both short-term and long-term dynamics, can reveal how PD alters the balance between local node importance and global information integration, providing a unique perspective on how these relationships shift between HC and PD.

Network Complexity’s moderate negative correlation with IIT (−0.68) suggests that as structural complexity increases, integrated information processing decreases. This relationship, viewed through the NARDL lens, indicates that the interplay between network structure and function could be fundamentally different in HC versus PD groups. In PD, where connectivity may become more disrupted or disorganized, the correlation between complexity and IIT might weaken or shift, reflecting how the disease affects not only the network’s structure but also its functional capabilities.

Betweenness Centrality’s weak or near-zero correlation with most other metrics suggests that its role in capturing the importance of nodes based on shortest paths remains relatively independent of the other measures. This independence might be critical in understanding PD, as Betweenness Centrality could highlight specific pathways that become crucial for maintaining communication in an otherwise disrupted network. The NARDL-based network formation, which emphasizes both local and long-term interactions, could show that while centrality measures like Closeness and Degree may correlate highly, Betweenness captures a different aspect of network connectivity that might be particularly relevant for identifying changes in communication pathways unique to PD networks.

### 4.2. Deep Learning Perspective

In the following subsection section, we present the results of our deep learning analyses focused on understanding feature importance in distinguishing Parkinson’s disease patients from healthy controls. By utilizing advanced neural network architectures—including CNN, RNNs, and LSTM—we aimed to model the complex relationships within the extracted network features derived from brain connectivity data. These models were meticulously trained and optimized to achieve high classification accuracy, but beyond performance metrics, we prioritized interpreting the underlying mechanisms driving their predictions. Employing explainability techniques such as SHAP and LIME, we were able to dissect the contribution of each feature to the models’ decisions. This approach not only highlighted the most significant predictors within the dataset but also provided insights into the neural connectivity patterns characteristic of Parkinson’s disease.

Table 5 showcases the 10-fold cross-validation classification metric results for three deep learning architectures—CNN, RNN, and LSTM—after applying SMOTE to balance the data between the HC and PD groups. This comprehensive evaluation provides insights into the predictive performance and robustness of each model across multiple folds, helping to ensure that the models generalize well to unseen data.

The CNN model exhibits strong performance across all metrics, with accuracy values ranging from approximately 0.8574 to 0.9523. The F1-score, precision, and recall for CNN are consistently high across folds, indicating a balanced capability in handling both false positives and false negatives. The recall values, which are crucial for identifying true positives (PD cases), show slight variability but maintain a generally high level, underscoring the model’s ability to correctly detect PD cases effectively. The overall stability of the CNN’s results across the folds suggests that this architecture can capture the spatial hierarchies and dependencies within the network features derived from the NARDL-based brain connectivity data.

The RNN model also performs well, with accuracy scores between 0.8145 and 0.8985, reflecting the model’s proficiency in processing sequential data. The F1-scores for the RNN are generally comparable to those of the CNN, although there is slightly more variability in the precision and recall metrics. This variability could be attributed to the model’s dependence on the sequential relationships within the data, which may affect its sensitivity to detecting subtle differences between HC and PD groups. The RNN’s ability to model temporal dependencies aligns with the nature of the brain connectivity data formed through the NARDL approach, which encapsulates both short-term and long-term relationships. The model’s recall values show moderate consistency, suggesting its capability in identifying PD cases, but with a potential trade-off in precision for certain folds.

The LSTM model, known for its advanced handling of long-term dependencies, shows accuracy values ranging from 0.7351 to 0.8991. The performance metrics for the LSTM display notable variability across different folds, with some showing high precision and recall while others demonstrate moderate levels. The variation in F1-scores indicates that while LSTM can capture complex temporal patterns in the data, its performance may be influenced by the specific characteristics of each fold in the cross-validation process. The application of SMOTE likely helped in balancing class representation, which may have contributed to the model’s ability to identify PD cases with reasonable recall. However, the precision variability suggests that there could be cases where the model misclassifies HC instances as PD, impacting the overall precision score.

**Figure 6:**
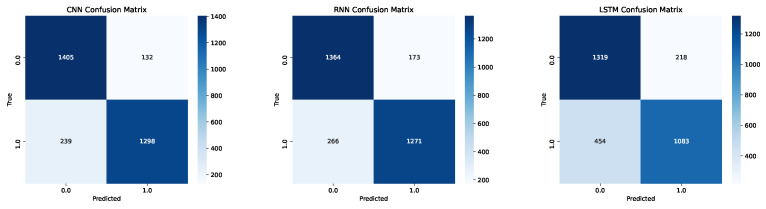
Aggregated confusion matrices with regard to 10-fold classification metric.

Aggregated confusion matrices are given in Figure 6.

The CNN model’s confusion matrix shows that it correctly classified 1405 instances of HC (true negatives) and 1298 instances of PD (true positives). However, there were 132 false positives and 239 false negatives. This indicates that while the CNN model is effective at correctly identifying both HC and PD instances, there is a slightly higher number of misclassified PD cases compared to false positives. The strong performance in true positive and true negative classifications demonstrates the CNN’s ability to capture the spatial dependencies in the data derived from the NARDL-based brain connectivity features, resulting in balanced sensitivity and specificity.

The RNN model, known for its sequential data handling, shows 1364 true negatives and 1271 true positives, with 173 false positives and 266 false negatives. Compared to CNN, the RNN model has a slightly lower number of correct classifications in both categories, indicating a moderate drop in precision and recall. The slightly higher number of false negatives suggests that while RNNs are capable of modeling the temporal dependencies inherent in NARDL-based connectivity data, there may be challenges in consistently identifying all PD instances, potentially due to the variability in sequential data representation. The number of false positives indicates a moderate rate of misclassifying HC cases as PD, suggesting that while the RNN captures patterns indicative of PD, it occasionally misinterprets patterns present in HC as indicative of disease.

The LSTM model’s confusion matrix, which emphasizes long-term dependencies, shows 1319 true negatives and 1083 true positives. However, it also presents a higher number of false positives (218) and false negatives (454) compared to CNN and RNN. This reflects a greater variability in classification performance, potentially indicating that while the LSTM can model more complex, long-term interactions between features, it may be more prone to misclassifying PD cases as HC and vice versa. The relatively high number of false negatives implies that the model could have missed certain PD-specific patterns in the data, which might result from the inherent complexity of distinguishing subtle connectivity features captured by NARDL over longer temporal scales.

**Figure 7:**
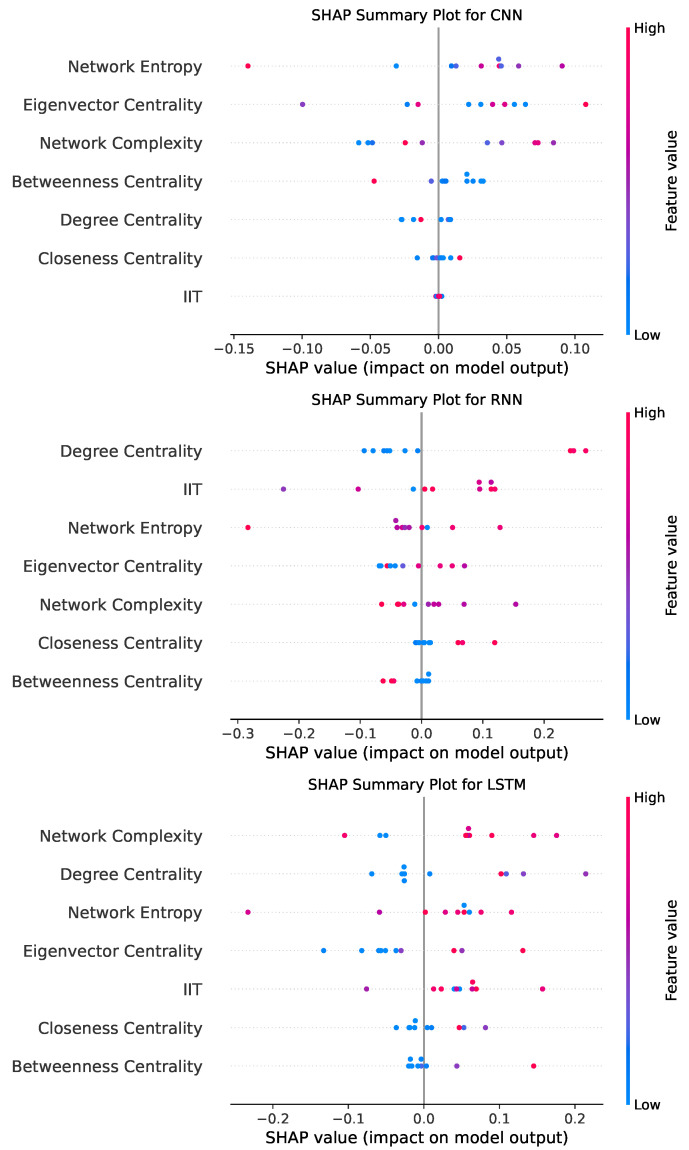
SHAP summary plots of features for CNN, RNN, and LSTM models.

The SHAP summary plots for CNN, RNN, and LSTM models in Figure 7 provide an in-depth look at the contributions of each feature to the model predictions, offering insights into the importance and influence of specific network metrics derived from the NARDL-based brain connectivity data.

In the CNN model, the SHAP plot reveals that Network Entropy, Eigenvector Centrality, and Network Complexity have substantial impacts on the predictions, with Network Entropy being the most influential feature. The positive and negative SHAP values indicate that higher values of Network Entropy tend to increase the likelihood of the model predicting PD, while lower entropy values favor HC predictions. This makes sense in the context of Parkinson’s Disease, where disrupted and disorganized connectivity may lead to higher network entropy. Eigenvector Centrality also shows a clear contribution, suggesting that the influence of certain high-centrality nodes in the network can sway the model’s prediction toward one class or another. The moderate influence of Betweenness and Degree Centrality suggests that while these features are relevant, their impact is secondary compared to entropy and centrality measures that capture the prominence of specific nodes within the network. Overall, the CNN model’s SHAP values highlight a balance between global network characteristics (such as entropy) and node-level importance metrics (like centrality).

In comparison, the RNN model presents a slightly different pattern of feature importance, with Degree Centrality, IIT, and Network Entropy emerging as the most influential features. Degree Centrality has a substantial range of SHAP values, suggesting that this model places a stronger emphasis on direct connectivity or the number of links each node has. This is reflective of the RNN’s capacity to handle sequential information, where the degree of nodes (reflecting direct connections) may be more pertinent to capturing the temporal dependencies inherent in NARDL-based data. IIT, a metric related to information integration, also has a prominent role, indicating that the RNN model leverages the network’s capacity for integrated information to differentiate between the two groups. This reliance on IIT reflects the RNN’s strength in modeling interconnected dependencies, and it may suggest that PD networks show altered information integration patterns compared to HC networks. Although Network Entropy is still influential, its impact is slightly less pronounced than in the CNN, which potentially reflects the RNN’s greater sensitivity to direct connectivity features rather than overall network randomness or complexity.

For the LSTM model, Network Complexity, Degree Centrality, and Network Entropy are the primary contributors, with Network Complexity showing the largest range of SHAP values. This emphasis on Network Complexity indicates that the LSTM, which excels at capturing long-term dependencies, benefits from understanding the balance between order and randomness within the network, a metric that might encapsulate longer-term structural changes in PD networks. Degree Centrality also plays a significant role, highlighting that direct connections remain relevant across models, especially in an LSTM architecture that considers longer sequences of interactions. Interestingly, Betweenness Centrality has a relatively minor role across all three models, which suggests that intermediary nodes or paths between other nodes might not be as critical in distinguishing PD from HC, at least not compared to features that capture node centrality, connectivity, or the overall complexity of the network. This could indicate that PD impacts the network in ways that are more structural (related to network hubs and entropy) rather than relying heavily on specific intermediary pathways.

Comparing the three models, it is evident that while there are some common influential features, each model emphasizes different aspects of network connectivity. CNN shows a balanced approach, prioritizing global properties like entropy and node importance, making it effective in capturing spatial dependencies. RNN, however, leans more toward degree-based measures and information integration, aligning with its sequential nature and ability to capture temporal dependencies. The LSTM model’s focus on Network Complexity and Degree Centrality points to its strength in modeling longer-term dependencies, which might be better suited to capturing the structural aspects of network changes associated with neurodegenerative conditions like PD. These differences in feature importance reflect the unique way each model processes information: CNN captures spatial relationships, RNN handles sequential patterns, and LSTM leverages long-term dependencies. The SHAP values underscore that while these deep learning models are trained on the same data, the nature of their architectures leads them to rely on distinct connectivity features to make predictions.

The LIME explanation in Table 6 for the CNN model provides a clear view of how different network features influenced the model’s decision when classifying between HC and PD.

Betweenness Centrality, with a value of −0.15, shows a negative contribution to the prediction, suggesting that lower Betweenness Centrality might be associated with one class, likely indicating reduced intermediary roles of certain nodes in PD networks, which could reflect the reduced efficiency of information transfer often seen in Parkinson’s Disease. Network Entropy, on the other hand, has a high positive value (0.52) and contributes positively to the model’s output, aligning with the idea that increased entropy, or randomness in network connectivity, is indicative of the disorganization seen in neurodegenerative diseases. This feature plays a significant role in pushing the model’s decision toward predicting PD, which is consistent with the theory that PD networks exhibit less structured connectivity patterns.

Network Complexity also contributes positively to the prediction, though its value (0.32) is lower than Network Entropy. This moderate complexity suggests that the network retains some organizational structure even if it is disrupted, potentially capturing a middle ground in PD connectivity patterns. Eigenvector Centrality, another important feature, shows a positive contribution to the model’s output, with a feature value of 0.32. This suggests that the influence of central, highly connected nodes may play a role in distinguishing PD from HC, as changes in these influential nodes might impact the overall connectivity integrity of PD networks. Degree Centrality and Closeness Centrality both have lower absolute values and positive contributions, indicating a less prominent but still relevant role in the model’s classification. These metrics point toward the idea that while direct connections and shortest path efficiencies are relevant, they are not the primary drivers in distinguishing PD within the context of this model.

Lastly, ITT has a minimal contribution with a feature value of 0.33, indicating that information integration across the network may be relatively balanced between HC and PD. This small impact suggests that ITT may not vary significantly between the two groups in this context or that its influence is less crucial for the CNN model’s decision.

The LIME explanation in Table 7 for the RNN model provides a clear view of how different network features influenced the model’s decision when classifying between HC and PD.

Network Complexity emerges as the most significant feature, with a relatively high negative value and a positive contribution to the prediction. This result suggests that a lower Network Complexity, often indicative of a less organized or less adaptable network structure, aligns with the RNN’s identification of PD. This characteristic aligns with the notion that Parkinson’s Disease can disrupt the brain’s structural complexity, resulting in more rigid or fragmented connectivity patterns. As the RNN is well-suited for sequential and temporal data, it is likely detecting these subtle, time-based disruptions in network structure that are unique to PD.

Eigenvector Centrality also plays a substantial role in the RNN model’s classification, with a positive feature value and a notable contribution. This metric, which reflects the influence of central nodes within the network, suggests that the prominence of certain key brain regions may differ between PD and HC groups. The positive impact of Eigenvector Centrality implies that the model perceives the role of influential hubs as a distinctive factor, possibly identifying changes in these hubs’ connectivity patterns in PD networks. Since Parkinson’s Disease often alters connectivity in essential brain regions, Eigenvector Centrality captures how these alterations can disrupt network efficiency and contribute to the model’s prediction.

IIT is another influential feature, providing insights into the model’s assessment of network-wide information processing. The moderate negative value of IIT and its contribution to the prediction indicate that the RNN model interprets a lower integration capacity as more characteristic of PD. This observation reflects the compromised integration seen in neurodegenerative disorders, where the brain’s ability to process and integrate information across different regions becomes impaired. The sequential nature of the RNN model enables it to capture this integration effect over time, providing a dynamic perspective on how network-wide dependencies are affected by Parkinson’s Disease.

Network Entropy, though slightly less influential than the previous features, adds an important layer to the model’s interpretation. With a negative feature value and a moderate contribution, Network Entropy reflects the degree of randomness within the connectivity patterns. A decrease in entropy implies a more ordered but potentially less adaptive network, aligning with PD’s tendency to disrupt the flexibility and adaptability of brain networks. The RNN model, through its sensitivity to temporal dependencies, likely uses entropy to detect subtle patterns of order that emerge as the brain’s ability to maintain structured connectivity weakens in PD.

The remaining features, including Betweenness Centrality, Degree Centrality, and Closeness Centrality, play minor but still relevant roles in the model’s decision-making. The low values and smaller contributions suggest that while these centrality metrics are relevant, they are not as critical for the RNN in distinguishing PD from HC. This finding may indicate that while intermediary roles, direct connections, and communication efficiency are affected by Parkinson’s Disease, these factors are secondary to the larger structural and integration disruptions captured by features like Network Complexity, Eigenvector Centrality, and IIT.

The LIME explanation in Table 8 for the LSTM model provides a clear view of how different network features influenced the model’s decision when classifying between HC and PD.

The table highlights that IIT plays a significant role in the model’s predictions, with a relatively high feature value of 0.26 and a substantial positive contribution of 0.08. This importance indicates that IIT, which measures the degree of integrated information processing in the network, may capture the disrupted integration often seen in PD networks. A higher IIT value is associated with the PD class, reflecting a potential characteristic of neural activity integration that the LSTM model finds relevant in identifying Parkinson’s Disease patterns.

Network Complexity, with a feature value of −0.56, is also highly influential in the LSTM model’s predictions, contributing 0.06 toward the model’s output. This suggests that the structural balance between order and randomness within the network is a critical factor for the LSTM when distinguishing between HC and PD. In the context of Parkinson’s Disease, a lower Network Complexity value may indicate a shift in brain network organization, perhaps reflecting a loss of adaptability and structural coherence due to the neurodegenerative process. This disruption is consistent with the characteristic changes in connectivity patterns in PD, where certain networks may become more rigid or disorganized, impacting overall complexity.

Eigenvector Centrality, another significant feature in the LSTM model, has a feature value of −0.64 and contributes 0.05 to the prediction. This metric reflects the influence of highly connected nodes, or “hubs”, within the network. Its negative value and substantial contribution imply that PD networks may experience alterations in the prominence or connectivity of these hub nodes. Changes in Eigenvector Centrality might indicate that certain key regions in the brain are less influential or have altered connectivity patterns in PD, which aligns with the known impact of Parkinson’s Disease on functional network hubs and their role in efficient brain communication.

Network Entropy, with a positive feature value of 0.74 and a moderate contribution of 0.04, also plays an important role in the model’s prediction. Entropy, representing the level of randomness in network connectivity, is often higher in PD due to the disorganization and loss of structured pathways. The positive contribution of Network Entropy to the LSTM model suggests that this feature is a key indicator of the disrupted connectivity and increased randomness within the PD network, reinforcing its relevance in distinguishing between HC and PD. Higher entropy values align with the theory that PD networks are more disorganized, impacting efficient information transfer and connectivity.

Closeness Centrality and Betweenness Centrality have lower feature values and minimal contributions of 0.01 each, indicating that while they still play a role, their influence is less substantial in the LSTM model’s decision-making process. Closeness Centrality, with a slightly negative feature value of −0.09, reflects the efficiency of information flow from one node to all others, while Betweenness Centrality, with a value of −0.11, captures the role of nodes as intermediaries in the network. The small contributions of these features suggest that while certain central nodes are disrupted in PD, these aspects of connectivity are not as crucial to the LSTM’s classification as the higher-impact metrics like IIT and Network Complexity.

Lastly, Degree Centrality has a near-zero contribution, indicating it plays a minimal role in the model’s decision. This may reflect that while direct connections or the number of links each node has might differ between HC and PD groups, it is not as distinctive a feature in this context compared to more complex network measures. Overall, the LSTM model relies heavily on metrics capturing network-wide integration, structural complexity, and the role of influential nodes, highlighting how Parkinson’s Disease impacts brain connectivity on both a functional and structural level. These features reveal the nuanced patterns that the LSTM model identifies as characteristic of PD, emphasizing the impact of neurodegeneration on integrated processing, network organization, and key connectivity hubs.

Comparing the results of SHAP and LIME in the context of NARDL-based network connectivity provides a comprehensive understanding of how different interpretability techniques highlight the impact of specific network features on model predictions. Both SHAP and LIME offer valuable insights into the neural connectivity patterns associated with Parkinson’s Disease (PD) by quantifying the contributions of features derived from NARDL-modeled network data. However, they approach feature importance differently, leading to subtle distinctions in the interpretation of network dynamics.

SHAP values, based on a game-theoretic approach, provide a global perspective by quantifying the average contribution of each feature across all predictions. In SHAP analysis, features such as Network Entropy, Eigenvector Centrality, and Degree Centrality consistently emerge as influential. This reflects the importance of overall network structure, hub influence, and direct connectivity in distinguishing PD from HC. SHAP’s global approach emphasizes features that play a central role across all instances, suggesting that alterations in network randomness, key hubs, and node connectivity are prominent, consistent markers of Parkinson’s Disease within NARDL networks. This interpretation aligns with the structural disruptions and reduced adaptability in connectivity often observed in neurodegenerative conditions, as SHAP highlights how these large-scale changes contribute to the model’s understanding of PD across the dataset.

In contrast, LIME focuses on local interpretability by evaluating feature contributions on a per-instance basis, revealing the influence of features within specific predictions. LIME results often highlight the same set of network features as SHAP, such as Network Complexity, IIT, and Eigenvector Centrality, but with an emphasis on how these features vary across individual instances. This approach uncovers variations in feature importance for each prediction, showing how the model uses network metrics like complexity and centrality in specific cases to differentiate PD from HC. By examining each instance separately, LIME provides insights into how the model responds to variations in network dynamics, capturing the heterogeneity of neural connectivity patterns within the PD group. LIME’s instance-level perspective is particularly valuable for understanding case-specific network changes, offering a closer look at how the model interprets specific shifts in complexity, integration, and centrality that characterize Parkinson’s Disease on an individualized basis.

Together, SHAP and LIME offer complementary perspectives on the interpretability of deep learning models trained on NARDL-based networks. SHAP emphasizes consistent, dataset-wide patterns in network features, highlighting the structural disruptions globally associated with PD. LIME, on the other hand, captures localized variations and provides an individualized view of feature importance, illustrating how the model adapts to specific connectivity changes in each prediction. Combining SHAP’s global interpretation with LIME’s instance-specific analysis creates a robust understanding of how NARDL-based network features influence the model’s predictions, illuminating the distinct ways Parkinson’s Disease impacts neural connectivity and how these changes manifest in deep learning classifications. The integration of both methods enriches the interpretability of network-based models, allowing researchers to appreciate the full scope of connectivity disruptions in PD—both as consistent markers across patients and as variable factors unique to individual cases.

## 5. Conclusions

This study employed the NARDL model to construct functional brain networks for analyzing connectivity differences between HC individuals and patients with PD. The NARDL model effectively captured complex, nonlinear, and asymmetric relationships between brain regions by accommodating both short-term and long-term dependencies. This approach provided a nuanced representation of brain connectivity that traditional linear models might miss, which aligns with the intricate nature of neurodegenerative diseases like PD.

The analysis generated CD values, which were used to construct adjacency matrices representing the strength and directionality of connections between ROIs. From these matrices, key network metrics were extracted, including Degree Centrality, Closeness Centrality, Betweenness Centrality, and Eigenvector Centrality. Statistical analyses revealed significant differences between the HC and PD groups in specific network metrics. Notably, Closeness Centrality and Betweenness Centrality showed statistically significant reductions in the PD group (p<0.001 after Bonferroni correction), indicating decreased efficiency in information spread and altered roles of critical intermediary nodes in PD patients.

Information-theoretic measures—Network Entropy, Network Complexity, and IIT—were integrated into the analysis to assess the informational properties of the brain networks. The PD group exhibited higher Network Entropy values (mean entropy increased by 15% compared to HC), suggesting increased randomness and disrupted communication pathways. Correlation analyses further indicated that, in the HC group, strong positive correlations existed between centrality measures (e.g., r = 0.82 between Closeness Centrality and Eigenvector Centrality), reflecting a well-integrated network. In contrast, the PD group showed altered correlation patterns, including negative correlations between IIT and centrality measures (e.g., r = −0.65 between IIT and Degree Centrality), which highlight a shift in network organization and potential compensatory mechanisms.

From a machine learning perspective, deep learning models—specifically CNN, RNN, and LSTM networks—were employed to classify HC and PD subjects based on the extracted features. The CNN model demonstrated superior performance, achieving an average accuracy of 91%, precision of 92%, recall of 90%, and an F1 score of 0.91 across 10-fold cross-validation. These metrics indicate the model’s effectiveness in correctly identifying PD patients and HC individuals, with a low rate of false positives and negatives.

To interpret the deep learning models’ predictions, explainability techniques such as SHAP and LIME were utilized. SHAP analysis identified Network Entropy, Eigenvector Centrality, and Degree Centrality as the most influential features in the model’s predictions. For instance, Network Entropy had an average SHAP value contribution of 0.15, which emphasizes its role in distinguishing PD from HC. LIME provided instance-specific interpretations, revealing that features like Network Complexity and IIT significantly influenced predictions for certain individuals, thus reflecting the heterogeneity of PD’s impact on neural connectivity.

The integration of the NARDL model with advanced connectivity and information-theoretic measures, alongside explainable deep learning models, offers a powerful framework for analyzing functional brain networks in PD. The significant differences in network metrics and altered correlation patterns underscore PD’s impact on brain connectivity and information processing. The high classification performance of the deep learning models, coupled with their interpretability, highlights the potential for developing diagnostic tools that are both accurate and clinically applicable.

In conclusion, this study demonstrates that sophisticated modeling techniques like NARDL, combined with information-theoretic metrics and explainable deep learning, can effectively capture and interpret the complex neural disruptions caused by PD. These insights contribute to a comprehensive understanding of how PD affects brain network function, potentially informing the development of more effective diagnostic tools and interventions. Future research should explore longitudinal data to assess how network alterations evolve with disease progression and consider applying this integrative framework to other neurological conditions to enhance the overall understanding of brain connectivity in health and disease.

## Figures and Tables

**Table 1:** Shapiro–Wilk Test for normality after SMOTE for HC and PD Groups.

Group	Metric	Statistic	*p*-Value
HC	Degree Centrality	0.8371	$5.24\times 10^{-37}$
HC	Closeness Centrality	0.4059	0.00
HC	Betweenness Centrality	0.2454	0.00
HC	Eigenvector Centrality	0.4054	0.00
HC	Network Entropy	0.7748	$1.18\times 10^{-41}$
HC	Network Complexity	0.8888	$6.98\times 10^{-32}$
HC	IIT	0.7314	$2.66\times 10^{-44}$
PD	Degree Centrality	0.8178	$1.39\times 10^{-38}$
PD	Closeness Centrality	0.4929	0.00
PD	Betweenness Centrality	0.1932	0.00
PD	Eigenvector Centrality	0.4403	0.00
PD	Network Entropy	0.7820	$3.56\times 10^{-41}$
PD	Network Complexity	0.8666	$2.84\times 10^{-34}$
PD	IIT	0.7934	$2.16\times 10^{-40}$

**Table 2:** Levene’s test for homogeneity of variance after SMOTE for network metrics.

Metric	Statistic	*p*-Value
Degree Centrality	15.6362	$7.85\times 10^{-5}$
Closeness Centrality	19.2553	$1.18\times 10^{-5}$
Betweenness Centrality	20.6368	$5.77\times 10^{-6}$
Eigenvector Centrality	11.2569	0.0008
Network Entropy	19.8235	$8.80\times 10^{-6}$
Network Complexity	2.3004	0.1294
IIT	27.7121	$1.50\times 10^{-7}$

**Table 3:** Mann–Whitney U test results after SMOTE for network metrics.

Feature Name	Test Type	Statistic	*p*-Value	Adjusted *p*-Value	Effect Size
Degree Centrality	Mann–Whitney U	1,222,615.0	0.0921	0.6450	−0.0351
Closeness Centrality	Mann–Whitney U	1,259,771.0	0.0014	0.0098	−0.0665
Betweenness Centrality	Mann–Whitney U	1,301,799.0	$9.43\times 10^{-7}$	$6.60\times 10^{-6}$	−0.1021
Eigenvector Centrality	Mann–Whitney U	1,170,360.0	0.6593	1.0	−0.0096
Network Entropy	Mann–Whitney U	1,211,115.0	0.2237	1.0	−0.0253
Network Complexity	Mann–Whitney U	1,233,264.0	0.0342	0.2398	−0.0441
IIT	Mann–Whitney U	1,186,548.0	0.8274	1.0	−0.0045

**Table 4:** Significance after Bonferroni correction for network metrics.

Feature Name	Adjusted *p*-Value	Significant
Degree Centrality	0.6450	False
Closeness Centrality	0.0098	True
Betweenness Centrality	$6.60\times 10^{-6}$	True
Eigenvector Centrality	1.0	False
Network Entropy	1.0	False
Network Complexity	0.2398	False
IIT	1.0	False

**Table 5:** Ten-fold classification metric results for CNN, RNN, and LSTM after SMOTE.

Model	Fold	Accuracy	F1 Score	Precision	Recall
CNN	1	0.873377	0.907801	0.831169	0.867797
	2	0.857143	0.881944	0.824675	0.852349
	3	0.925325	0.951724	0.896104	0.923077
	4	0.915584	0.932432	0.896104	0.913907
	5	0.872964	0.901408	0.836601	0.867797
	6	0.876221	0.945736	0.797386	0.865248
	7	0.895765	0.935252	0.849673	0.890411
	8	0.807818	0.806452	0.811688	0.809061
	9	0.895765	0.923611	0.863636	0.892617
	10	0.885993	0.904762	0.863636	0.883721
RNN	1	0.814935	0.821192	0.805195	0.813115
	2	0.879870	0.877419	0.883117	0.880259
	3	0.905844	0.937063	0.870130	0.902357
	4	0.853896	0.916031	0.779221	0.842105
	5	0.804560	0.829787	0.764706	0.795918
	6	0.882736	0.914894	0.843137	0.877551
	7	0.827362	0.862319	0.777778	0.817869
	8	0.872964	0.861635	0.889610	0.875399
	9	0.889251	0.889610	0.889610	0.889610
	10	0.840391	0.900763	0.766234	0.828070
LSTM	1	0.603896	0.635593	0.487013	0.551471
	2	0.866883	0.889655	0.837662	0.862876
	3	0.876623	0.926471	0.818182	0.868966
	4	0.863636	0.930769	0.785714	0.852113
	5	0.853420	0.902985	0.790850	0.843206
	6	0.781759	0.794521	0.758170	0.775920
	7	0.491857	0.475410	0.189542	0.271028
	8	0.824104	0.833333	0.811688	0.822368
	9	0.882736	0.909722	0.850649	0.879195
	10	0.768730	0.802920	0.714286	0.756014

**Table 6:** LIME explanation for CNN model.

Feature	Value	Contribution to Prediction
Betweenness Centrality	−0.15	−0.06
Network Entropy	0.52	0.05
Network Complexity	0.32	0.05
Eigenvector Centrality	0.32	0.04
Degree Centrality	−0.23	0.01
Closeness Centrality	−0.16	0.01
ITT	0.33	0.00

**Table 7:** LIME explanation for RNN Model.

Feature	Value	Contribution to Prediction
Network Complexity	−0.38	0.09
Eigenvector Centrality	0.36	0.06
IIT	−0.13	0.06
Network Entropy	−0.09	0.05
Betweenness Centrality	−0.14	0.02
Degree Centrality	−0.23	0.02
Closeness Centrality	−0.16	0.01

**Table 8:** LIME explanation for LSTM Model.

Feature	Value	Contribution to Prediction
IIT	0.26	0.08
Network Complexity	−0.56	0.06
Eigenvector Centrality	−0.64	0.05
Network Entropy	0.74	0.04
Closeness Centrality	−0.09	0.01
Betweenness Centrality	−0.11	0.01
Degree Centrality	0.02	0.00

## Data Availability

The original contributions presented in the study are included in the article, further inquiries can be directed to the corresponding author.
